# Redescription of *Hargeria rapax* (Harger, 1879) and description of *H. chetumalensis* a new species from the Mexican Caribbean (Crustacea, Peracarida, Tanaidacea, Leptocheliidae) based upon morphological and molecular evidence

**DOI:** 10.7717/peerj.7472

**Published:** 2019-08-19

**Authors:** Jani Jarquín-González, Luis F. Carrera-Parra

**Affiliations:** 1 Departamento de Sistemática y Ecología Acuática, El Colegio de la Frontera Sur, Chetumal, Quintana Roo, México; 2 Departamento de Ciencias y Humaninades, Universidad de Quintana Roo, Cozumel, Quintana Roo, México

**Keywords:** Taxonomy, COI-barcoding, Morphology, Cryptic species, New species

## Abstract

Until now, *Hargeria* was considered a monospecific leptocheliid genus, with the species *Hargeria rapax* considered a taxon with a wide distribution, from the Northwestern Atlantic to the Mexican Caribbean. Herein, after a detailed revision of type and topotype materials and specimens collected from the Mexican Caribbean, a new species *H. chetumalensis* sp. nov. is described, and the redescription of *H. rapax* is provided. Also, we found a significant genetic divergence between the two species based on the nucleotide sequences of cytochrome oxidase subunit I, which support the morphological data. The morphological features used to recognize both species are also adequate to link males, females, and juvenile stages, although these species have a high intraspecific polymorphism.

## Introduction

In 1879, Oscar Harger described a new species of tanaidacean from Annisquam, Massachusetts, USA, *Leptochelia rapax*; unfortunately, the description lacks illustrations. The females of this species were distinguished from other leptocheliids by the last segment of the antennule, which is scarcely longer than the preceding one; while the males, by having the elongate and slender antennules and chelipeds (Harger, 1879). Later, Harger (1880) gave a new characterization for this species and provided drawings of the cheliped and full body of the male. Much later, this species was transferred to a new genus, *Hargeria*, by Lang (1973) because the males have a unique feature within the tanaidaceans, an anal plate, as well as by the presence of two pointed spines on the maxilliped endite in females. Furthermore, Lang (1973) drew some of the female’s appendages; however, no accompanying description was provided.

For over 30 years, *Hargeria* Lang, 1973 was considered a monospecific genus. Nevertheless, Larsen & Wilson (2002), using phylogenetic methods based on morphological data, found that *Hargeria rapax* and *L. dubia* (Krøyer, 1842) formed a sister-group relationship. This relationship between *Hargeria* and *Leptochelia* was later corroborated by Bird & Larsen (2009) and also regarded *Hargeria* as a synonym of *Leptochelia*. Likewise, Drumm (2010), based on molecular data (COI, H3, and 28S), tested the hypothesis that *H. rapax* is the sister species of *L. dubia* and suggested that *Hargeria* should be considered a junior synonym of *Leptochelia* since both genera can only be differentiated by presence of the anal plate in males of *Hargeria* that is absent in *Leptochelia*.

We point out that the results and conclusions above described were obtained using a very limited number of leptocheliids species because those analyses were done with the main task of elucidating the phylogeny of higher taxa (superfamily Paratanaidoidea by Larsen & Wilson, 2002, and Bird & Larsen, 2009, or Tanaidacea by Drumm, 2010) not only the Leptocheliidae. Recently, Guţu (2016) reestablished *Hargeria* based on an extensive morphological revision of numerous taxa belonging to Leptocheliidae; he found that *Hargeria* can be recognized from other leptocheliid genera by having some particular features: both sexes have merus of pereopod 1 with one ventrodistal and one dorsodistal seta; merus of pereopods 2 and 3 with two ventrodistal setae; uropodal endopod five-articulate; females with two flat spines and one distal seta on maxilliped endite; the presence of an anal plate in the male, as well as the presence of a small proximal process, and a large distal process on the fixed finger of the male cheliped.

In the context of geographic distribution, *H. rapax* is an estuarine species that has been considered with a wide distribution along the Northeastern Atlantic coast from Massachusetts, USA to Chetumal Bay, Mexico (Suárez-Morales et al., 2004; García-Madrigal, Heard & Suárez-Morales, 2005). Herein, we provide a detailed redescription of male and female of *H. rapax* based on type and topotypical materials. Also, we describe the male and female of a new species of *Hargeria* based on morphological and molecular evidence. This species represents the first tanaidacean described from the Mexican Caribbean.

## Materials and Methods

We studied type and topotypical specimens of *H. rapax* deposited in the collections of the National Museum of the Natural History, Smithsonian Institution, Washington (USNM); also, specimens identified as *H. rapax* deposited in the Reference Collection of Benthos (ECOSUR) of El Colegio de la Frontera Sur, Chetumal, Mexico were reviewed.

The tanaids were examined under a stereomicroscope Carl Zeiss SV6. The total length of specimens was measured from the anterior end of the cephalothorax to the posterior margin of the pleotelson. Dissection of appendages was generally performed on the right side of the body; the pieces were mounted in glycerol and sealed with transparent nail varnish. Drawings of taxonomically features were made with a camera lucida at 4–40×. In the description section, we follow Larsen (2003), Bird (2011, 2012), and Guţu (2016) for the general morphology and setal terminology. For morphological analysis of setal and cuticular structures of adult (female and male), juvenile, and manca specimens were processed to be observed by SEM. Specimens were dehydrated in a series of different concentration of hexamethyldisilazane. Once air-dried, they were mounted on aluminum stubs and coated with gold for observation using a JEOL JSM-6010Plus-LA scanning electron microscopy at the Scanning Electron Microscopy Laboratory (LMEB), ECOSUR-Chetumal.

For molecular analysis, we use the whole organism. DNA barcoding was carried out at the Canadian Center for DNA Barcoding (University of Guelph), following the standard protocols of the program “Barcode of the Life.” Cytochrome oxidase subunit I (COI) nucleotide sequences between 579–658 bp were amplified using M13F (5′-TGTAAAACGACGGCCAGT-3′) and M13R (5′-CAGGAAACAGCTATGAC-3′) primers (Messing, 1983). Sequence data, electropherograms, trace files, primer details, photographs, life stages, and collection localities for specimens are available within the project “Tanaidacea from Mexico” at Barcode of Life Data System. Some sequences of *H. rapax*, *L. forresti* (Stebbing, 1896), *L. longichelipes* (Lang, 1973), and *Chondrochelia dubia* (Krøyer, 1842) (as *L. dubia*) were obtained from the GenBank database (Table 1).

**Table 1:** Specimens included in molecular analyses.

Species	Locality	BOLD system # process ID	GenBank accession no.	Reference
*H. chetumalensis* sp. nov.	Guerrero Lagoon, Q. Roo, Mexico	TANAI104-15 TANAI105-15 TANAI106-15 TANAI107-15 TANAI109-15		
	Raudales, Q. Roo, Mexico	TANAI071-15 TANAI072-15 TANAI073-15 TANAI074-15 TANAI075-15 TANAI076-15 TANAI077-15 TANAI078-15 TANAI079-15 TANAI110-15 TANAI111-15 TANAI112-15 TANAI113-15 TANAI115-15 TANAI116-15 TANAI117-15		Current work
*H. rapax*	Dania Beach, FL, USA		HM016214	Drumm (2010)
	Indian River Lagoon, FL, USA		KP254749	Leray & Knowlton (2015)
	Sheepshead Cove, MD, USA		KU905783 KU905876	R Aguilar et al., unpublished data
	White Marsh Creek, MD, USA		KU906052	
*C. dubia*	Fort Lauderdale, FL, USA		HM016215	Drumm (2010)
*L. forresti*	Dania Beach, FL, USA		HM016206	Drumm (2010)
*L. longichelipes*	Belize		HM016201	Drumm (2010)

All sequences were aligned using ClustalW method; the selection of the best model of substitution was determined according with the lowest Bayesian information criterion score. As result, we used Hasegawa–Kishino–Yano, using a discrete Gamma distribution (+G) with five rate categories and by assuming that a certain fraction of sites is evolutionarily invariable (+I) as model to construct a tree using the maximum likelihood analysis. Furthermore, we used the Kimura 2-Parameter model to estimate the average evolutionary divergence over sequence pairs within and between species. All analyses were carried out with Mega 7 (Kumar, Stecher & Tamura, 2015).

The electronic version of this article in portable document format will represent a published work according to the International Commission on Zoological Nomenclature (ICZN), and hence the new names contained in the electronic version are effectively published under that Code from the electronic edition alone. This published work and the nomenclatural acts it contains have been registered in ZooBank, the online registration system for the ICZN. The ZooBank LSIDs (Life Science Identifiers) can be resolved and the associated information viewed through any standard web browser by appending the LSID to the prefix http://zoobank.org/. The LSID for this publication is: urn:lsid:zoobank.org:pub:B55A046E-55AA-491A-ABED-C9BA3D7300DE. The online version of this work is archived and available from the following digital repositories: PeerJ, PubMed Central, and CLOCKSS.

## Systematics

Superorder Peracarida Calman, 1904
Order Tanaidacea Dana, 1849
Suborder Tanaidomorpha Sieg, 1980
Family Leptocheliidae Lang, 1973
Subfamily Leptocheliinae Lang, 1973

**Genus *Hargeria*Lang, 1973**
*Hargeria* Lang, 1939: 225.

**Type species.** *Leptochelia rapax* Harger, 1879, by original designation (Lang, 1973).

**Diagnosis.** Amended from Lang (1973). **Both sexes.** Pereopods 2 and 3 with two dorsodistal setae and one ventrodistal spine on propodus. Pereopod 4 with three distal spines and two setae on carpus. Uropodal endopod with five articles; exopod small, unarticulate. **Males.** With an anal plate longer than pleotelson. Antennule almost as long as body. Cheliped slender, slightly longer than body; carpus five to eight times as long as broad; fixed finger with one small proximal process and one large distal process on cutting edge. **Females.** Maxillipedal endite with three flat spines, the subdistal smaller.

**Figure 1:**
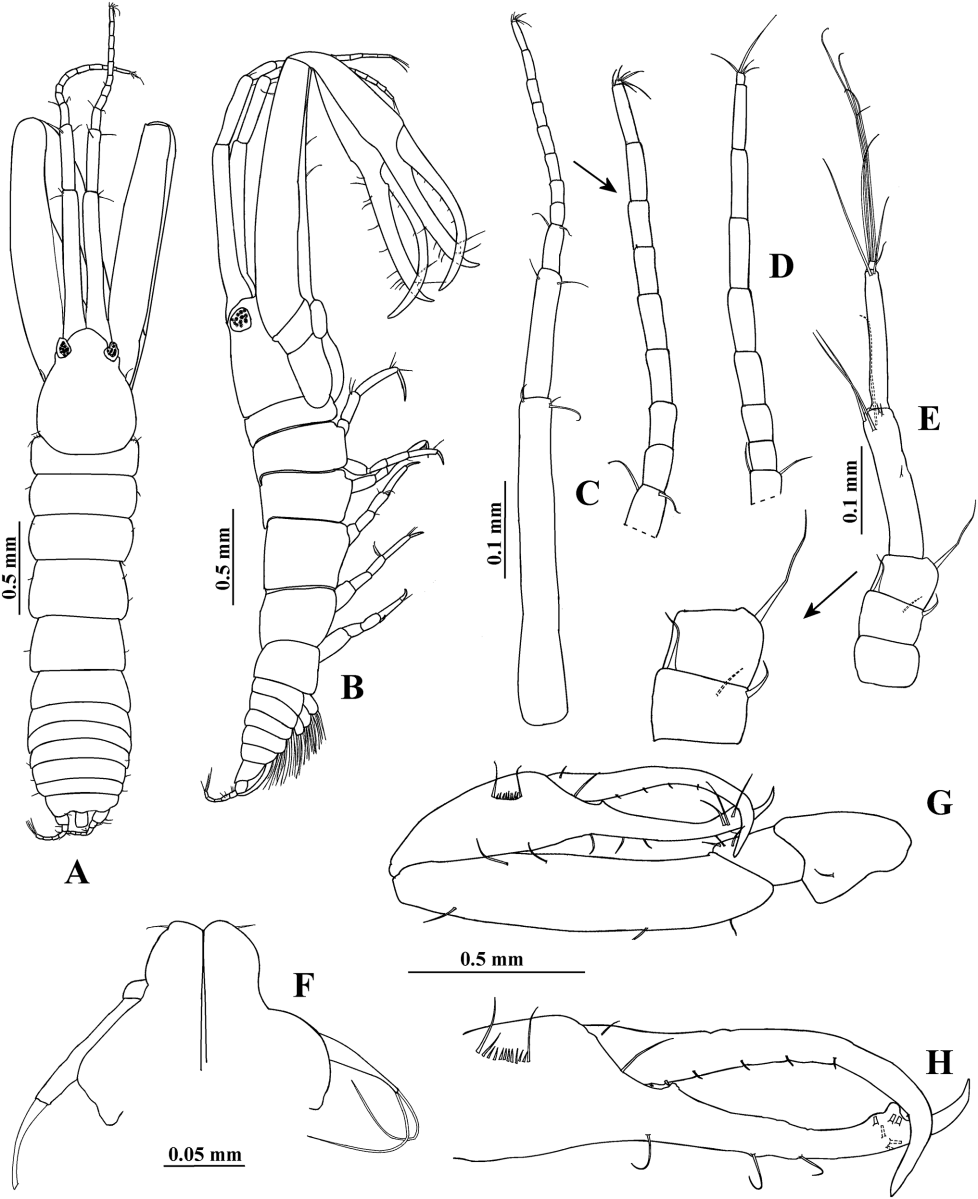
*Hargeria rapax*, type and topotype specimens. (A) Habitus. Topotype USNM 65779, adult male, 2.5 mm. (B) Habitus. (C) Antennule. (E) Antenna. (F) Maxilliped and maxillules. (G) Cheliped closed. (H) Detail of chelipedal cutting edge. Topotype USNM 101781, adult male, 2.3 mm. (D) Detail of antennular flagellum; asthetasc were omitted for clarity.

**Figure 2:**
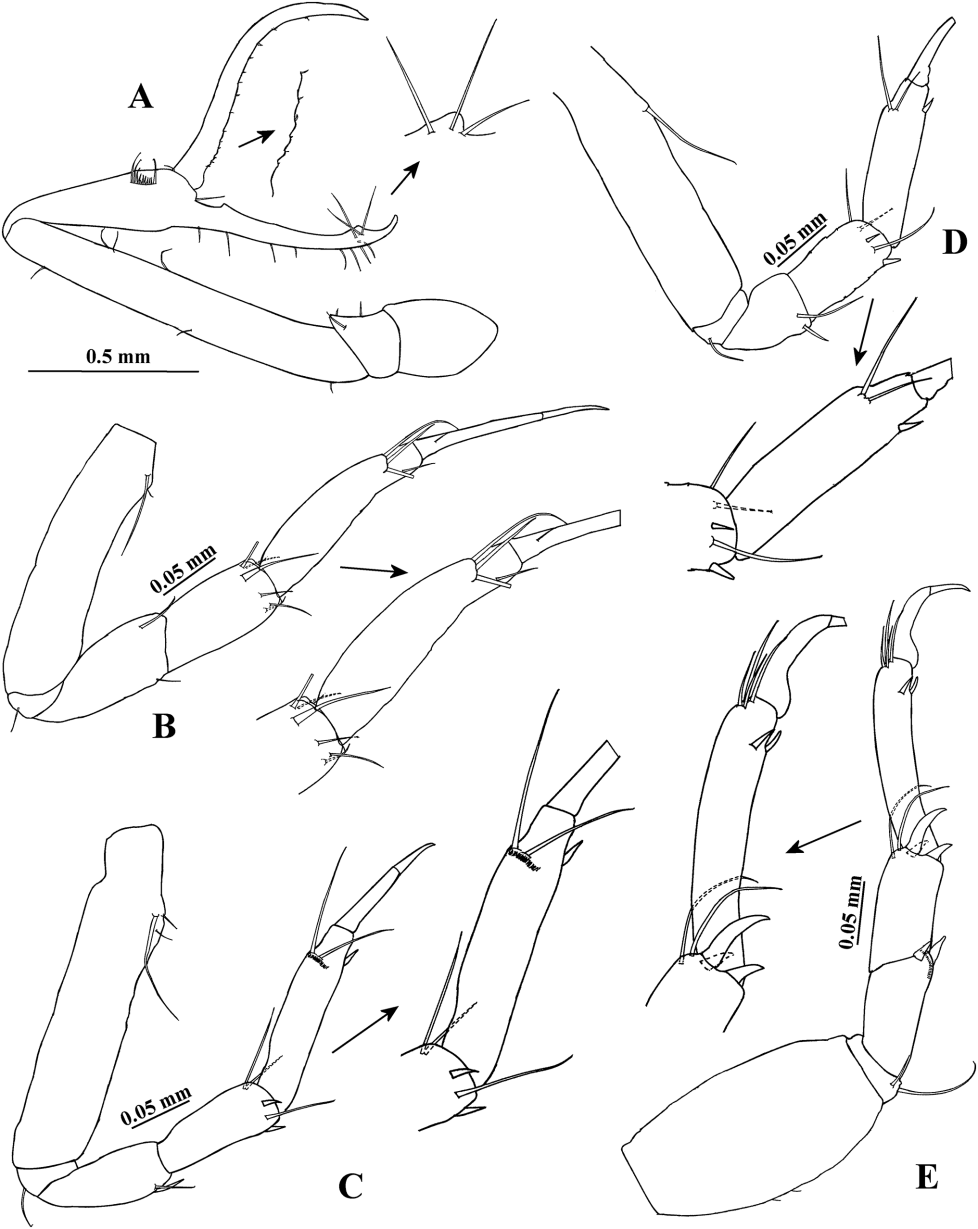
*Hargeria rapax*, topotype specimens. Adult male, 2.3 mm, USNM 101781. (A) Cheliped open. Adult male, 2.5 mm, USNM 65779. (B) Pereopod 1. (C) Pereopod 2. (D) Pereopod 3. (E) Pereopod 4.

**Figure 3:**
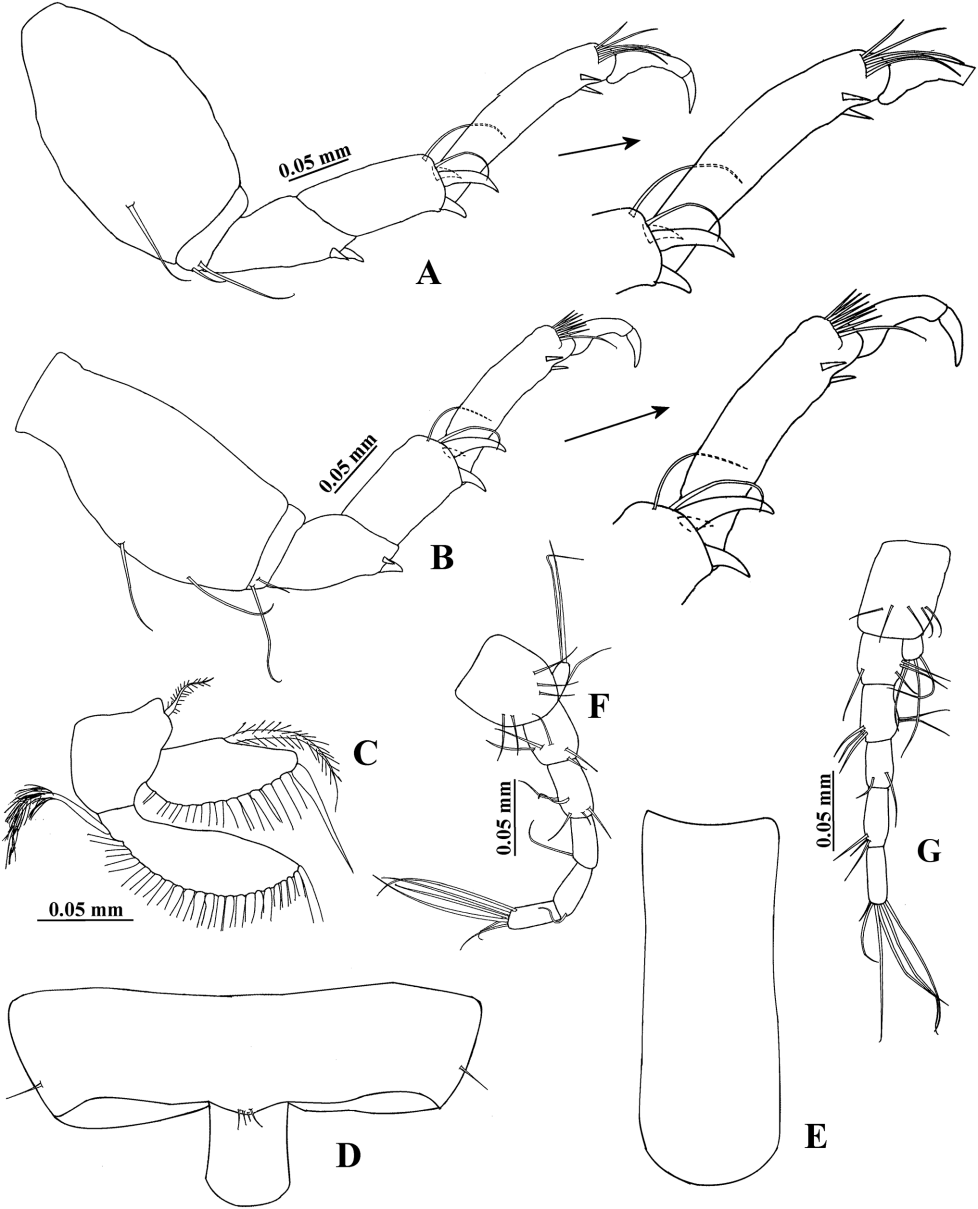
*Hargeria rapax*, topotype specimens. Adult male, 2.5 mm, USNM 65779. (A) Pereopod 5. (B) Pereopod 6. (C) Pleopod 1. (D) Pleotelson and anal plate. (E) Anal plate. (F) Uropod. Adult male, 2.3 mm, USNM 101781. (G) Uropod.

**Figure 4:**
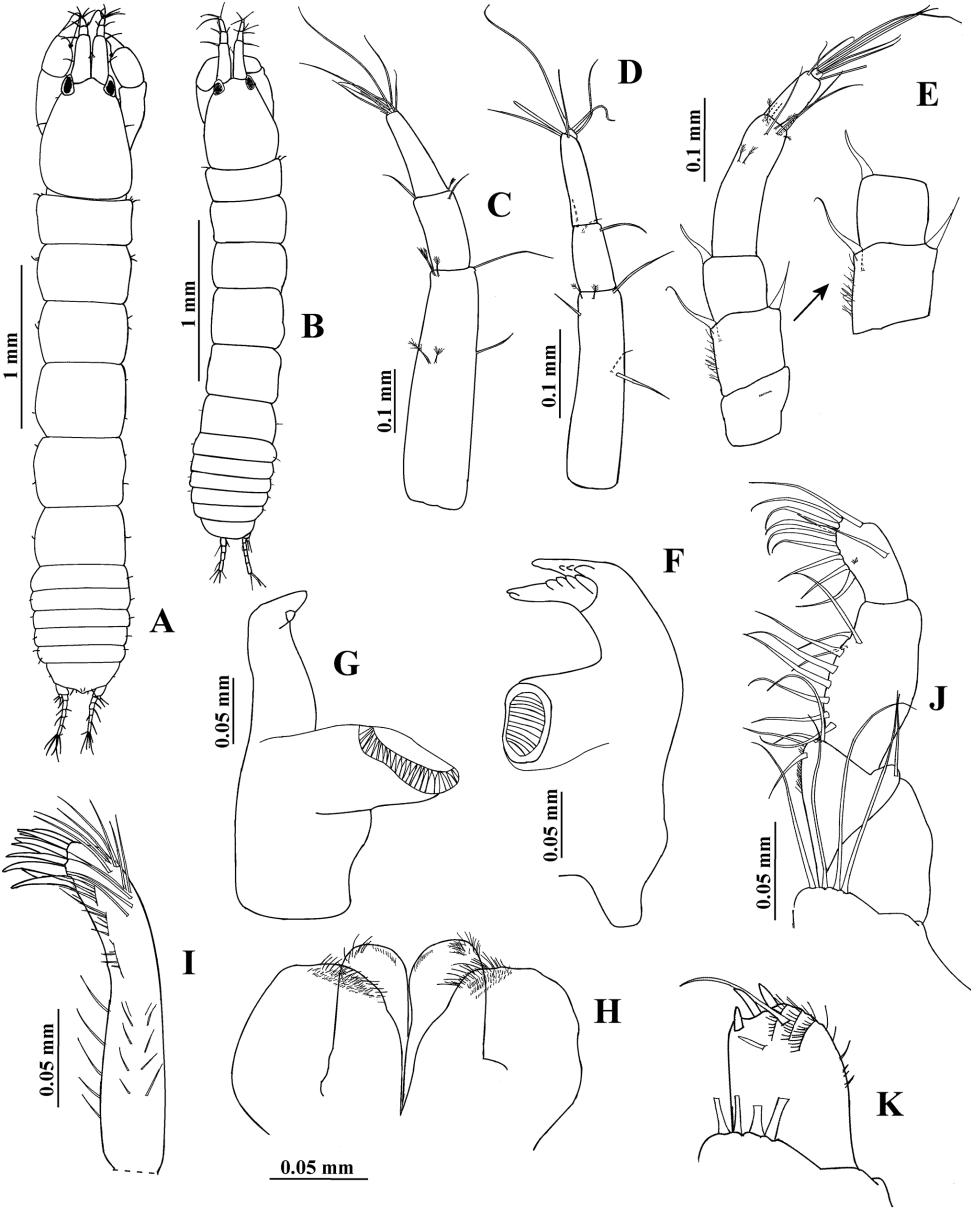
*Hargeria rapax*, topotype specimens. Non-ovigerous female, 3.7 mm, USNM 65779. (A) Habitus. (C) Antennule. (E) Antenna. (F) Left mandible. (G) Right mandible. (H) Labium. (I) Maxillule. (J) Maxillipedal palp. (K) Maxillipedal endite. Ovigerous female, 2.9 mm, USNM 101781. (B) Habitus. (D) Antennule.

**Figure 5:**
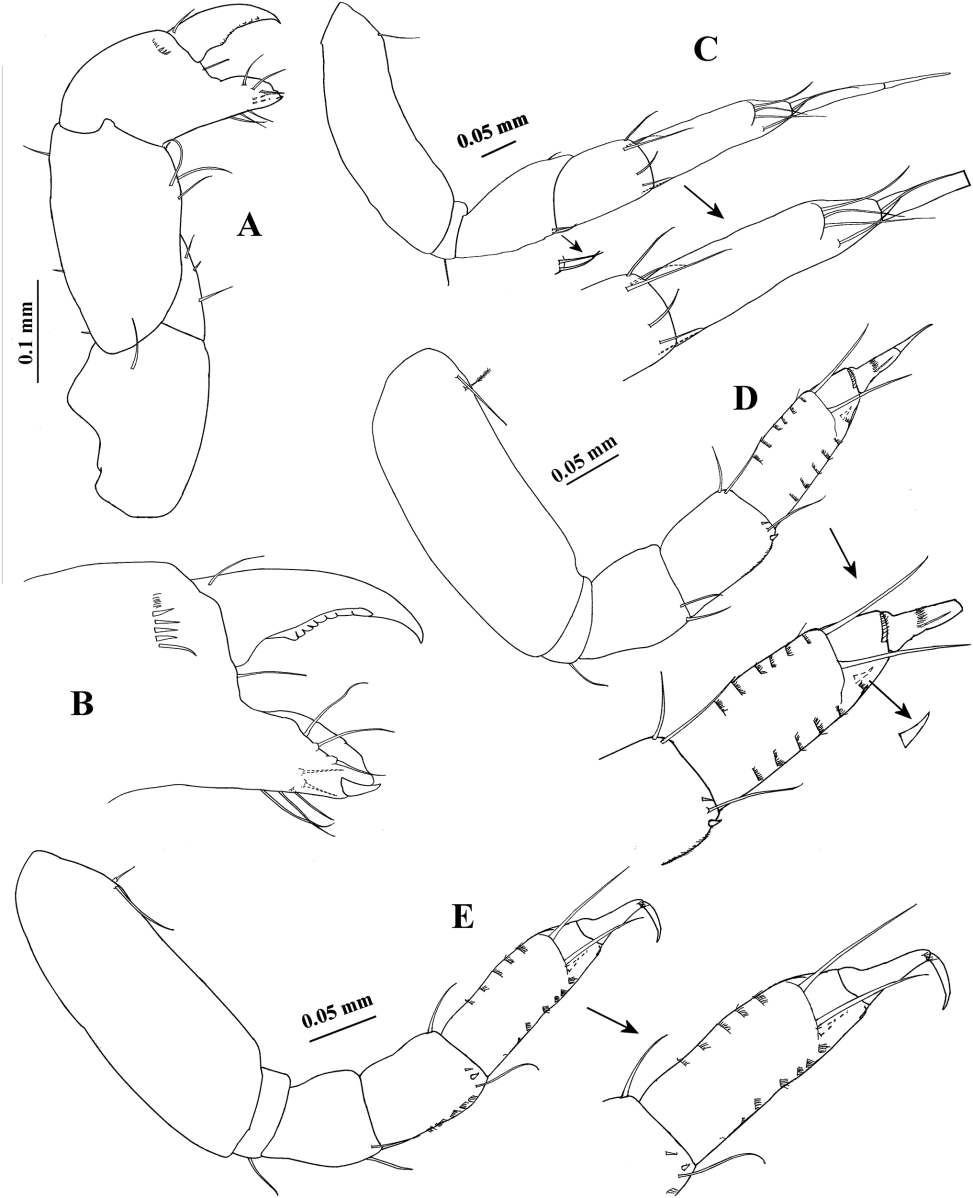
*Hargeria rapax*, topotype specimen USNM 65779. Non-ovigerous female, 3.7 mm. (A) Cheliped. (B) Detail of cutting edge of cheliped. (C) Pereopod 1. (D) Pereopod 2. (E) Pereopod 3.

**Figure 6:**
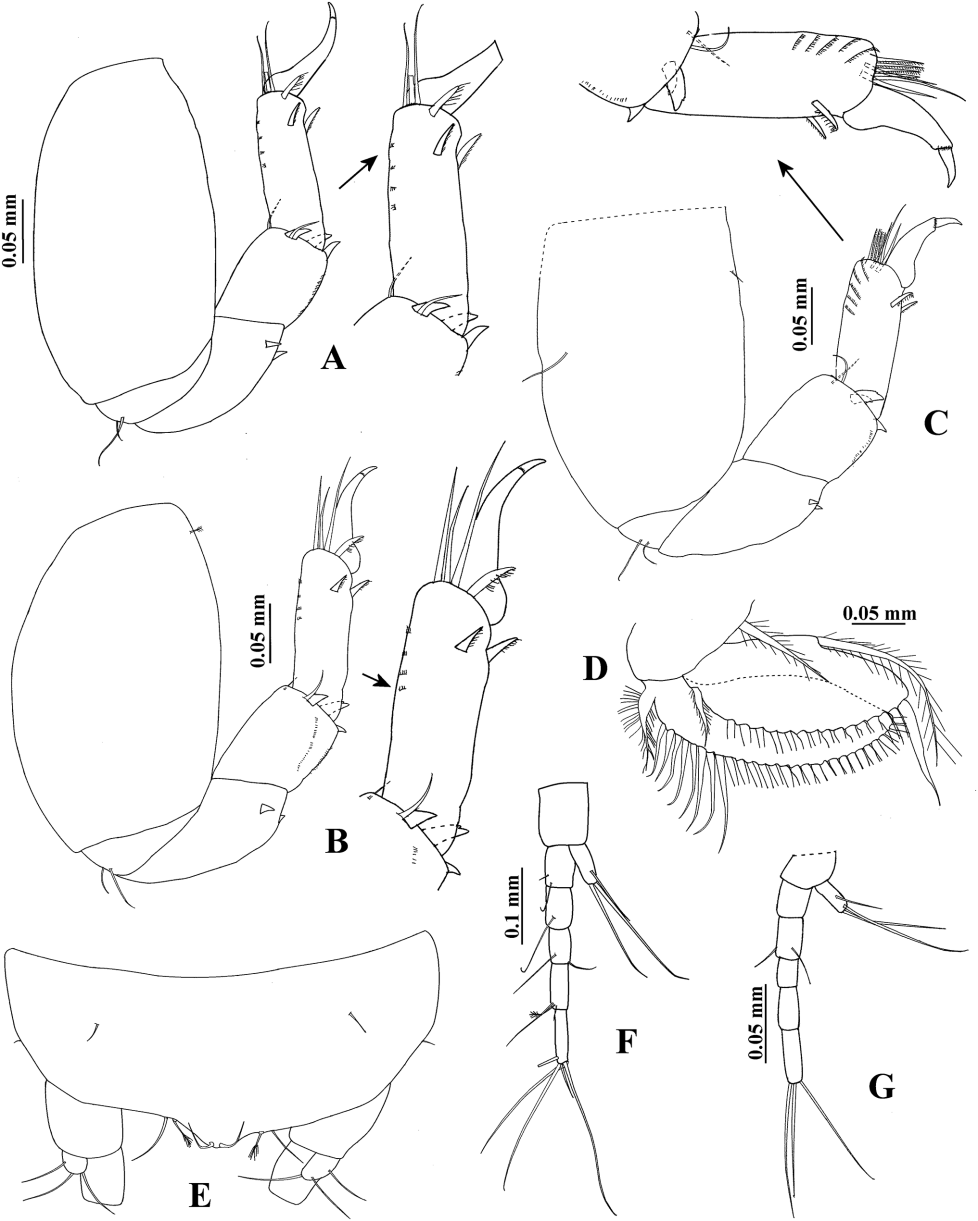
*Hargeria rapax*, topotype specimens USNM 65779. Non-ovigerous female, 3.7 mm. (A) Pereopod 4. (B) Pereopod 5. (C) Pereopod 6. (D) Pleopod 1. (E) Pleotelson. (F) Uropod. Ovigerous female, 2.9 mm. (G) Uropod.

***Hargeria rapax*** **(Harger, 1879)**
Figures 1–6
*Leptochelia rapax:* Harger, 1879: 163–164; Harger, 1880: 424–426, figs. 89–90.
*Hargeria rapax*: Lang, 1973: 202; Sieg, 1983: 422–424; Guţu, 2016: 19, 22, 27, 32, 56–57.

**Type material.** Syntypes, two males, USNM 32176, Annisquam, Massachusetts, USA, in muddy bottom, four m, coll. Gardiner, August 1879.

**Topotypical material.** USNM 101781, one ovigerous female, two non-ovigerous females, three males, Pocasset River, Cape Cod, Massachusetts, USA, in seagrass and algae, coll. Burbank, W. D., June 16, 1958. USNM 65779, nine ovigerous females, 26 non-ovigerous females, 11 males, Potomac River, Cobb Island, Maryland, USA, in algae, coll. Cochran, D. M., June 21, 1931.

**Diagnosis. Male.** Rostrum convex, broad, extending beyond eyes. Anal plate capitate, less than three times as long as broad, do not exceed the length of peduncle and first article of uropodal endopod combined. Cheliped carpus five to seven times as long as broad; fixed finger with pointed proximal process on cutting edge. Pereopod 1 with two distal setae on merus; with six distal setae on carpus. Pereopod 4 with two long distal setae that do not exceed the median length of propodus. **Female.** Right mandible with incisor smooth and pointed apex. Maxillipedal endite with three flat spines (pointed terminally). Pereopod 1 with one dorsodistal setae and two ventrodistal setae on merus.

**Description. Adult male**. Topotype USNM 65779, 2.5 mm (Figs. 1B and 3D–3E). Body 5.2 times as long as broad. Cephalothorax oval, longer than broad, slightly longer than the first three pereonites combined. Rostrum convex, broad, exceeding beyond eyes. Pereon about 2.5 times as long as cephalothorax; pereonites 1 and 6 narrower than other pereonites; pereonite 4 broader than other pereonites. Pleon less than 0.2 times the body length. Pleotelson about 0.3 times as long as pleon, with two dorsolateral setae; posterior end with four small dorsodistal setae. Anal plate capitate, less than three times as long as broad, do not exceed the length of peduncle and first article of uropodal endopod combined.

Antennule (Fig. 1C) 0.7 times as long as total body length, first peduncle article longer than cephalothorax, about seven times as long as broad, with three distal setae (one of them small). Second peduncle article about 0.4 times as long as first peduncle article, 5.4 times as long as broad, with two distal setae. Third peduncle article about 0.1 times as long as first peduncle article, 2.5 times as long as broad, with two distal setae. Flagellum 0.6 times as long as first peduncle article; with nine articles; first eight articles longer than broad, with two or three aesthetascs (not illustrated); ninth article minute, with five distal setae.

Antenna (Fig. 1E). First article as long as broad, naked. Second article 0.7 times as long as broad, with two distal setiform spines and one simple seta. Third article 0.8 times as long as broad, with one dorsodistal setiform spine. Fourth article 4.7 times as long as broad, with six setae on distal half (three of them small). Fifth article 0.9 times as long as fourth article, about eight times as long as broad, with two distal setae. Sixth article minute, with six distal setae.

Mouthparts reduced. Maxilliped (Fig. 1F) rudimentary, with two proximal setae; maxillule palps with two setae.

Cheliped (Figs. 1G–1H) 1.3 times as long as total body length. Basis 1.5 times as long as broad, with one dorsolateral seta. Merus longer than broad, with three ventrodistal setae. Carpus about five times as long as broad, with two ventrodistal setae and three dorsal setae. Propodus with seta near dactylus articulation; comb-row of 13 setae. Fixed finger with two processes on cutting edge, proximal small, triangular, with pointed apex, distal long, tubular, with blunt apex; with nine setae, six of them situated on ventral side. Dactylus with one proximal seta and seven ventral minute spines.

Pereopod 1 (Fig. 2B) with basis 4.1 times as long as broad, with two dorsoproximal setae, one of them small. Ischium with one seta. Merus 1.2 times as long as carpus, 2.4 times as long as broad, with two distal setae. Carpus twice as long as broad, with six distal setae. Propodus 3.9 times as long as broad, with distal setae (three dorsal and one ventral). Dactylus and unguis combined as long as propodus. Dactylus with one proximal seta.

Pereopod 2 (Fig. 2C) with basis 4.9 times as long as broad, with three dorsoproximal setae, two of them small. Ischium with one seta. Merus as long as carpus, 2.4 times as long as broad, with distoventral seta and spine. Carpus 2.4 times as long as broad, with three distal setae and two ventrodistal spines. Propodus 4.6 times as long as broad, with two dorsodistal setae, one ventrodistal spine and scales. Dactylus and unguis combined about 0.6 times as long as propodus, naked.

Pereopod 3 (Fig. 2D) similar to pereopod 2, but basis four times as long as broad. Merus 1.4 times as long as broad. Carpus 1.7 times as long as broad. Propodus 3.2 times as long as broad.

Pereopod 4 (Fig. 2E) with basis 1.8 times as long as broad, with two small ventral setae. Ischium with two setae. Merus as long as carpus, twice as long as broad, with two distal spines and scales. Carpus 2.1 times as long as broad, with three distal spines and two long setae that do not exceed the median length of propodus. Propodus 5.3 times as long as broad, with four dorsodistal setae and two ventrodistal spines. Dactylus and unguis combined about 0.5 times as long as propodus, naked.

Pereopod 5 (Fig. 3A) similar to pereopod 4, but basis 1.6 times as long as broad, with one ventrodistal seta. Propodus with five dorsodistal setae.

Pereopod 6 (Fig. 3B) similar to pereopods 4 and 5, but basis 1.9 times as long as broad, with two ventral setae. Carpus 1.9 times as long as broad. Propodus 4.3 times as long as broad, with eight dorsodistal setae.

Pleopod 1 (Fig. 3C) with one circumplumose dorsal seta on peduncle. Exopod with one proximoventral circumplumose seta and 21 ventral setae. Endopod with one circumplumose dorsal seta and 13 ventral setae.

Uropod (Fig. 3F) with five dorsal setae on peduncle. Exopod 0.6 times as long as first article of uropodal endopod, with three setae. Endopod first article broader than other articles; first and second article with five distal setae; third article smaller than other articles, with one distal seta; fourth and fifth articles subequal; fifth article with six distal setae.

**Non-ovigerous female**, Topotype USNM 65779, 3.7 mm (Fig. 4A). Body 6.2 times as long as broad. Cephalothorax oval, 1.3 times as long as broad, 0.7 times as long as first three pereonites combined. Pereon three times as long as cephalotorax; pereonites 2 and 3 subequal in length; pereonite 4 longer than other pereonites. Pleon about 0.2 times as long as body length. Pleotelson (Fig. 6E) 0.3 times as long as pleon, with four dorsolateral setae; posterior end rounded, slightly expanded, with four distal setae and two small plumose sensory setae.

Antennule (Fig. 4C) with four articles. First article 3.7 times as long as broad, with two setae and four plumose sensory setae on distal half. Second article 0.3 times as long as first article, 1.9 times as long as broad, with two distal setae and one plumose sensory seta. Third article 0.4 times as long as first article, three times as long as broad, with two distal setae and one aesthetasc. Fourth article minute, with four distal setae.

Antenna (Fig. 4E). First article 1.2 times as long as broad, with scales. Second article slightly longer than broad, with two distal spines and small distolateral setae. Third article as long as broad, with one distal spine. Fourth article 3.3 times as long as broad, with three setae and plumose sensory setae on distal half. Fifth article 0.4 times as long as fourth article, 2.2 times as long as broad, with two distal setae. Sixth article minute, with five distal setae.

Mouthparts. Left mandible (Fig. 4F) with incisor shorter than lacinia mobilis, with three small processes; lacinia mobilis with four small processes and bifurcate apex; molar cylindrical, with triturative ridges. Right mandible (Fig. 4G) with incisor smooth and pointed apex; molar thick, with triturative ridges. Labium (Fig. 4H) distally setose, with outer lobe shorter but broader than inner lobe. Maxillule (Fig. 4I) setose, with nine spines. Maxilliped (Fig. 4J) with four long setae on basis; first palp article 1.2 times as long as broad, naked; second palp article 1.5 times as long as broad, with one dorsodistal seta, four ventrodistal seta and small lateral setae; third palp article 1.9 times as long as broad, with nine ventral setae; fourth palp article 2.1 times as long as broad, with 10 ventral setae, one dorsolateral seta and scales; endite (Fig. 4K) with three flat spines (pointed terminally), the subdistal smaller, one dorsodistal seta, coupling hook, lateral setae and scales.

Cheliped (Figs. 5A and 5B) 0.2 times as long as total body length. Basis 1.4 times as long as broad, with one dorsal seta. Merus longer than broad, with three ventral setae. Carpus 1.8 times as long as broad, with three ventrodistal setae and three dorsal setae. Propodus with seta near dactylus articulation; comb-row with five setae. Fixed finger with cutting edge raised, with eight setae on distal half. Dactylus with proximodorsal seta and eight lamellae on ventral edge.

Pereopod 1 (Fig. 5C) with basis 3.6 times as long as broad, with one proximal seta. Ischium with one seta. Merus 1.2 times as long as carpus, 1.6 times as long as broad, with small setae (one dorsal, two ventral). Carpus 1.5 times as long as broad, with six distal setae. Propodus 3.2 times as long as broad, with distal setae (three dorsodistal and one ventral). Dactylus and unguis combined as long as propodus; dactylus with one proximal seta.

Pereopod 2 (Fig. 5D) with basis 2.5 times as long as broad, with one proximal seta and one plumose sensory seta. Ischium with one seta. Merus as long as carpus, 1.3 times as long as broad, with two distoventral setae. Carpus 1.5 times as long as broad, with three setae, two ventrodistal minute spines and scales. Propodus 2.5 times as long as broad, with two dorsodistal setae, small distoventral spine and scales. Dactylus and unguis combined 0.6 times as long as propodus; dactylus with subdistal seta and scales.

Pereopod 3 (Fig. 5E) similar to pereopod 2, but basis 2.7 times as long as broad. Propodus 2.4 times as long as broad.

Pereopod 4 (Fig. 6A) with basis 1.8 times as long as broad, naked. Ischium with two setae. Merus 1.7 times as long as carpus, with two ventrodistal spines. Carpus 1.2 times as long as broad, with two distal setae, three spines and scales. Propodus 3.2 times as long as broad, with three dorsodistal setae, one pinnate seta, two ventrodistal serrate spines and scales. Dactylus and unguis combined 0.7 times as long as propodus; dactylus and unguis joint with scales.

Pereopod 5 (Fig. 6B) similar to pereopod 4.

Pereopod 6 (Fig. 6C) similar to pereopods 4 and 5, but basis with small dorsal seta and one ventroproximal seta. Merus twice times as long as broad. Propodus 2.7 times as long as broad, with three dorsodistal simple setae, four pinnate setae, and two ventrodistal serrate spines.

Pleopod 1 (Fig. 6D) with one circumplumose dorsal seta on peduncle. Exopod with one proximoventral circumplumose seta and 28 ventral setae. Endopod with one dorsal circumplumose seta, one ventroproximal circumplumose seta, and 15 ventral setae.

Uropod (Fig. 6F) with naked peduncle. Exopod uniarticulate, as long as first article of endopod, with three setae. Endopod with five articles, first two articles with subequal length, first article with two setae, second article with one seta; third article shorter than other articles, with two setae; fourth article longer than other articles, with two setae and one plumose sensory seta; fifth article more slender than other articles, with five setae.

**Variability**. Males. Syntype USNM 32176, 2.6 mm. Body (Fig. 1A) 4.5 times as long as broad. Topotype USNM 101781, 2.3 mm. Antennule (Fig. 1D) with eight flagellar articles; cheliped (Fig. 2A) with carpus about seven times as long as broad, dactylus with 14 min spines, fixed finger with 11 setae, eight of them situated on ventral side; first article of uropodal endopod with four setae (Fig. 3G). Ovigerous female. Topotype USNM 101781, 2.9 mm (Fig. 4B). Antennule (Fig. 4D), first article with four setae and two plumose sensory setae on distal half; third article 0.5 times as long as first article; maxilliped with three long setae on basis, with six ventral setae on third palp article, with eight ventral setae on fourth article of palp; pereopod 1 with four distal setae on carpus; second article of uropodal endopod with two setae (Fig. 6G).

**Distribution**. Northwestern Atlantic, from Massachusetts to Florida.

**Habitat**. Shallow-water, in muddy bottom and sandy bottom with seagrass and algae.

**Remarks**. Historically, *H. rapax* has been considered a species with a wide distribution, from the Northwestern Atlantic to the Mexican Caribbean. However, the morphological and molecular analysis of specimens from Mexican Caribbean, have shown that they differ from *H. rapax* (see below remarks section of *H. chetumalensis* sp. nov.).

*Hargeria rapax*, as noted above, was regarded as a junior synonym of *Leptochelia* based on phylogenetic studies where the sister relationships between *H. rapax* and *L. dubia* (currently *C. dubia*) was observed. Recently, Guţu (2016) reestablished it based on a morphological revision of Leptocheliidae. Herein, we agree with this idea by the following reasons; the male of *Hargeria* is mainly characterized by having an anal plate, which is absent in *Leptochelia* and *Chondrochelia* Guţu, 2016. Also, the cheliped fixed finger of *Hargeria* has a small proximal process and a large distal process on cutting edge, whereas in *Leptochelia* can be observed only a medial process and in *Chondrochelia* one large proximal process and one small distal process. Moreover, *Hargeria* has uropodal exopod uniarticulate, while in *Leptochelia* it is biarticulate and in *Chondrochelia* uni- or biarticulate. In *Hargeria* the length of cheliped carpus is up to 8.0 times as long as broad, whereas in *Leptochelia* the carpus is more than eight times as long as broad; *Hargeria* has uropodal endopod five-articulate and the first two articles without long setae, while *Leptochelia* has uropodal endopod six-articulate and the first two articles with long setae.

Regarding females, the maxillipedal endite of *Hargeria* has three flat spines (the subdistal smaller); while *Leptochelia* only has two flat spines and *Chondrochelia* three to five flat spines. Other differences between these genera can be seen in propodus of Pereopods 2 and 3. For example, although *Hargeria* and *Leptochelia* have the same setation on propodus of Pereopod 2 (two dorsodistal setae and one ventrodistal spine), they differ on propodus of Pereopod 3, *Hargeria* has two dorsodistal setae and one ventrodistal spine, whereas *Leptochelia* has one dorsodistal seta and one ventrodistal spine. A similar condition occurs when compare *Hargeria* with *Chondrochelia*; both genera have two dorsodistal setae and one ventrodistal spine on propodus of Pereopod 3, but they differ because *Hargeria* has two dorsodistal setae and one ventrodistal spine on propodus of Pereopod 2, while *Chondrochelia* has three dorsodistal setae and one ventrodistal spine. Furthermore, these morphological differences are supported by the results of the molecular analysis (see below).

***Hargeria chetumalensis* sp. nov.**
urn:lsid:zoobank.org:act:A8D8DCA7-E71C-4124-83B7-F5E66CDA31A1
(Figures 7–14)
*Hargeria rapax*: Carrera-Parra, González & Salazar-Vallejo, 1997: 61–64; Suárez-Morales et al., 2004: 47–53, figs. 21–24; García-Madrigal, Heard & Suárez-Morales, 2005: 1165. (non Harger, 1879).

**Type material**. Holotype adult male, ECOSUR 0209, 2.9 mm, Raudales, 18°42′25.2″N 88°15′18″W, Quintana Roo, Mexico, in *Bathophora* sp. on rock, 0.5 m, May 28, 2015. Paratypes: 13 ovigerous females ECOSUR 0210, 44 non-ovigerous females ECOSUR 0211, 17 adult males ECOSUR 0212, same data as holotype.

**Additional material.** ECOSUR-C1079, five ovigerous females, 10 non-ovigerous female, three males, Calderitas, 18°29′13.2″N 87°45′25.2″W, Chetumal Bay, Quintana Roo, Mexico, in *Bathophora* sp. on rocks, June 28, 2016. ECOSUR-C1080, 20 ovigerous females, 32 non-ovigerous females, nine males, mouth of Guerrero Lagoon, 18°42′30.4″N 88°09′05.2″W, Quintana Roo, Mexico, in *Bathophora* sp. on rocks, April 23, 2016.

**Molecular material.** ECOSUR TANAI104-15, TANAI105-15, TANAI106-15, TANAI107-15, TANAI109-15 (one manca, two juvenile females, two non-ovigerous females): Guerrero Lagoon, 18°41′19.3″N 88°15′50.4″W, Quintana Roo, Mexico, in *Bathophora* sp. on rocks, 0.5 m, May 28, 2015. ECOSUR TANAI071-15, TANAI072-15, TANAI073-15, TANAI074-15, TANAI075-15, TANAI076-15, TANAI077-15, TANAI078-15, TANAI079-15, TANAI110-15, TANAI111-15, TANAI112-15, TANAI113-15, TANAI115-15, TANAI116-15, TANAI117-15 (two mancae, four juvenile females, four non-ovigerous females, two ovigerous females, two juvenile males, two adult males): Raudales, 18°42′25.2″N 88°15′18″W, Quintana Roo, Mexico, in *Bathophora* sp. on rocks, 0.5 m, May 28, 2015.

**Diagnosis. Male.** Rostrum pointed, narrow, not extending beyond eyes. Anal plate tongue-shaped, more than times as long as broad, extending beyond length of peduncle and first article of uropodal endopod combined. Cheliped with carpus about eight times as long as broad; fixed finger with blunt proximal process on cutting edge. Pereopod 1 with one small ventrodistal serrate seta on merus; with three distal serrate setae on carpus. Pereopod 4 with two long distal serrate setae extending beyond the median length of propodus. **Female.** Right mandible with incisor crenulate and slightly bifurcate apex. Maxillipedal endite with three flat spines (two of them rounded terminally, the smaller pointed). Pereopod 1 with one small ventrodistal serrate seta on merus.

**Etymology.** The specific epithet is derived from the locality name of the Chetumal Bay System, where the species was found.

**Figure 7:**
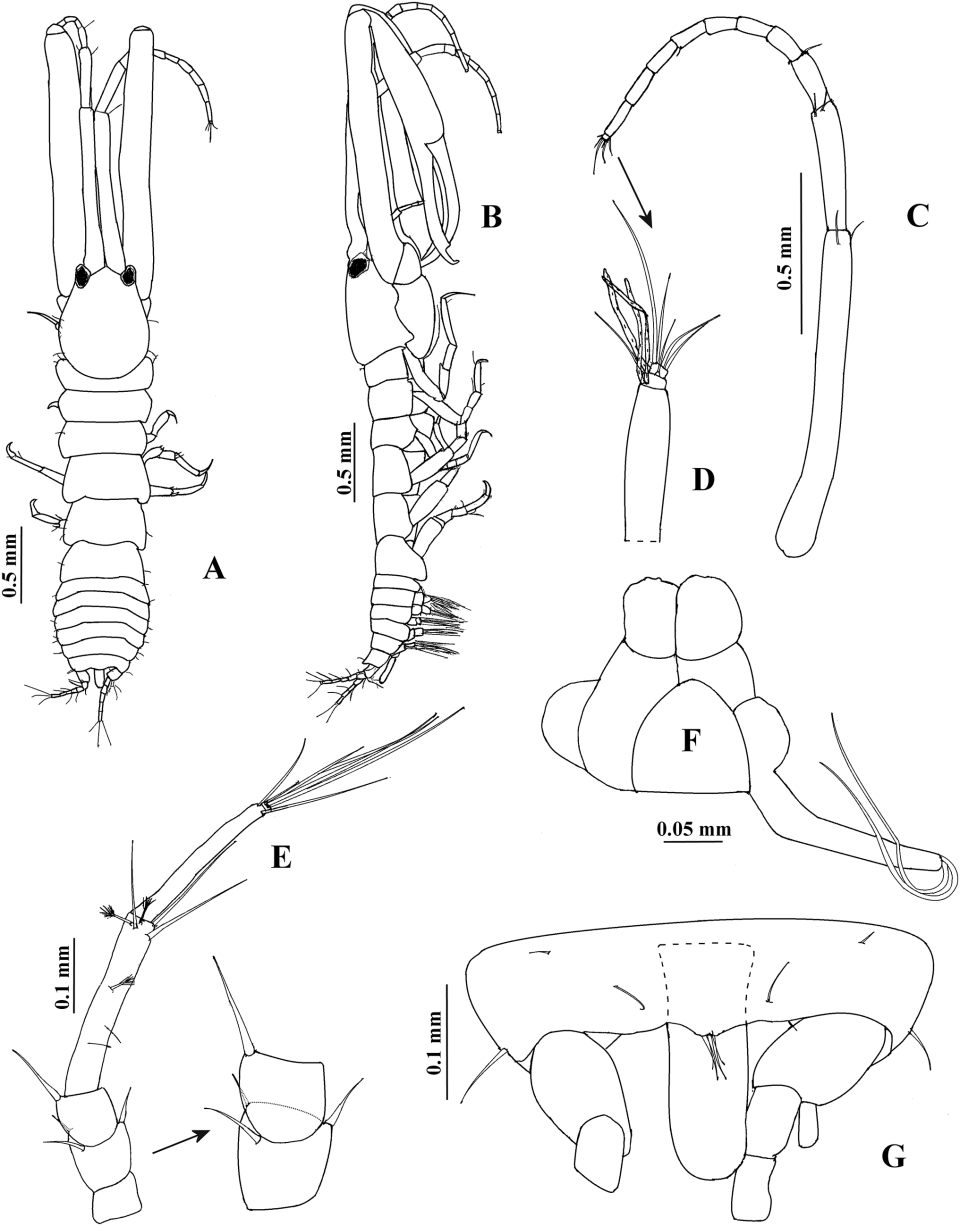
*Hargeria chetumalensis* sp. nov. Holotype ECOSUR0209, adult male, 2.9 mm. (A) Habitus, dorsal view. (B) Habitus, lateral view. (C) Antennule, (D) Distal article, antennule. (E) Antenna. (F) Maxilliped and maxillules. (G) Pleotelson with anal plate.

**Description. Adult male.** Holotype ECOSUR0209, 2.9 mm (Figs. 7A–7B and 7G). Body 4.2 times as long as broad. Cephalothorax oval, 1.3 times as long as broad, 0.8 times as long as first three pereonites combined. Rostrum pointed, narrow, not extending beyond eyes. Pereon about 2.8 times as long as cephalothorax; pereonite 1 shorter than other pereonites; pereonites 2, 3, and 6 with subequal length, pereonites 4 and 5 with subequal length. Pleon about 0.2 times as long as total body length. Pleotelson about 0.2 time as long as pleon, with eight dorsolateral setae; posterior end with four small dorsodistal setae. Anal plate (Fig. 9C) tongue-shaped, more than three times as long as broad, extending beyond length of peduncle and first article of uropodal endopod combined.

Antennule (Figs. 7C and 7D) 0.9 times as long as total body length; first peduncle article 1.5 times as long as cephalothorax, more than eight times as long as broad, with two distal setae; second peduncle article 0.4 times as long as first peduncle article, 5.4 times as long as broad, with two distal setae; third peduncle article 0.2 times as long as first peduncle article, 2.8 times as long as broad, with two distal setae. Flagellum 0.8 times as long as first article, with nine articles; first seven articles longer than broad, each with 1–2 aesthetascs; last two articles smallest; last article with three distal setae.

Antenna (Fig. 7E). First article about 0.8 times as long as broad, naked. Second article as long as broad, with two distal setiform spines and one simple seta. Third article 0.9 times as long as broad, with one dorsodistal setiform spine. Fourth article 6.6 times as long as broad, with two small subproximal setae, three simple setae and three plumose sensory setae on distal half. Fifth article 0.9 times as long as fourth article, 8.5 times as long as broad, with two distal setae. Sixth article minute, with five distal setae.

Mouthparts reduced. Maxilliped rudimentary (Fig. 7F), maxillule palp with two setae.

**Figure 8:**
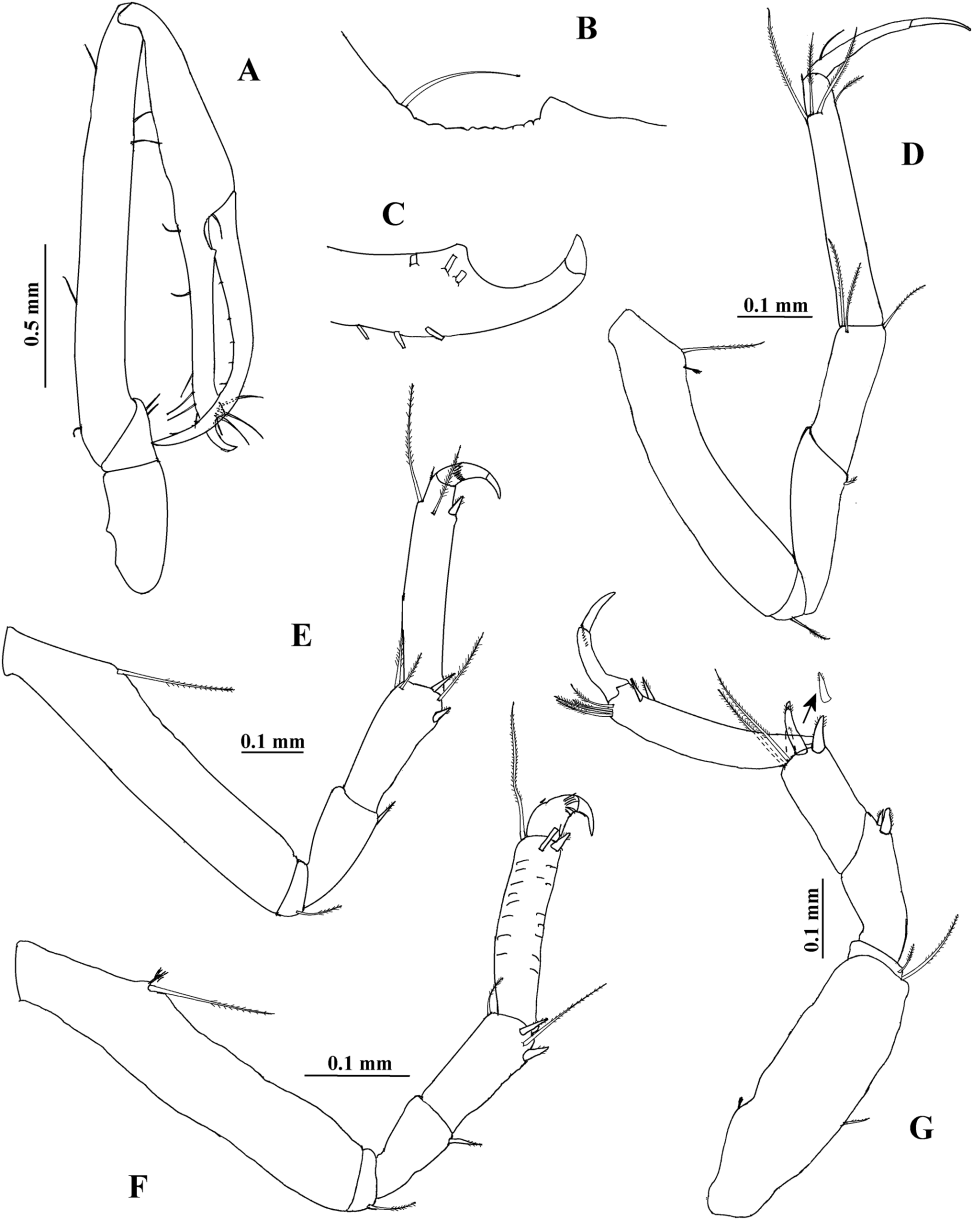
*Hargeria chetumalensis* sp. nov. Holotype ECOSUR0209, adult male, 2.9 mm. (A) Cheliped. (B) Proximal chelipedal process. (C) Distal chelipedal process. (D) Pereopod 1. (E) Pereopod 2. (F) Pereopod 3. (G) Pereopod 4.

Cheliped (Figs. 8A–8C) 1.6 times as long as total body length. Basis 2.1 times as long as broad, naked. Merus longer than broad, with two ventrodistal setae. Carpus eight times as long as broad, with two subdistal ventral setae and three dorsal setae. Propodus with seta near dactylus articulation, comb-row with 24 setae (not illustrated). Fixed finger with two processes on cutting edge; proximal small, triangular, striate, with blunt apex; distal long, tubular, striate, with blunt apex; distally crenulate, with ten setae, six of them situated on ventral side. Dactylus with ten ventral minute spines.

Pereopod 1 (Fig. 8D) with basis 5.1 times as long as broad, with one proximal serrate seta and one small plumose sensory seta. Ischium with one serrate seta. Merus 1.2 times as long as carpus, 3.1 times as long as broad, with one small ventrodistal serrate seta. Carpus 3.2 times as long as broad, with three serrate setae. Propodus 5.6 times as long as broad, with distal serrate setae (three dorsal and one ventral). Dactylus and unguis combined 0.8 times as long as propodus; dactylus with one dorsoproximal seta.

Pereopod 2 (Fig. 8E) with basis 5.5 times as long as broad, with one dorsoproximal serrate seta. Ischium with one serrate seta. Merus 0.9 times as long as carpus, twice as long as broad, with one ventrodistal serrate spine. Carpus 2.5 times as long as broad, with three distal serrate setae and two ventrodistal serrate spines. Propodus 4.9 times as long as broad, with two dorsodistal serrate setae and one ventrodistal serrate spine. Dactylus and unguis combined 0.3 times as long as propodus; dactylus with subdistal scales.

Pereopod 3 (Fig. 8F) similar to pereopod 2, but carpus 2.1 times as long as broad and with two distal serrate setae.

Pereopod 4 (Fig. 8G) with basis 3.2 times as long as broad, with one proximodorsal small plumose sensory seta and one mesioventral serrate seta. Ischium with two unequal serrate setae. Merus 1.2 times as long as carpus, 2.6 times as long as broad, with two distal serrate spines. Carpus 2.4 times as long as broad, with three spines and two long distal serrate setae extending beyond median length of propodus. Propodus 5.5 times as long as broad, with four dorsodistal serrate setae and two ventrodistal serrate spines. Dactylus and unguis combined about 0.5 times as long as propodus; dactylus and unguis joint with scales.

**Figure 9:**
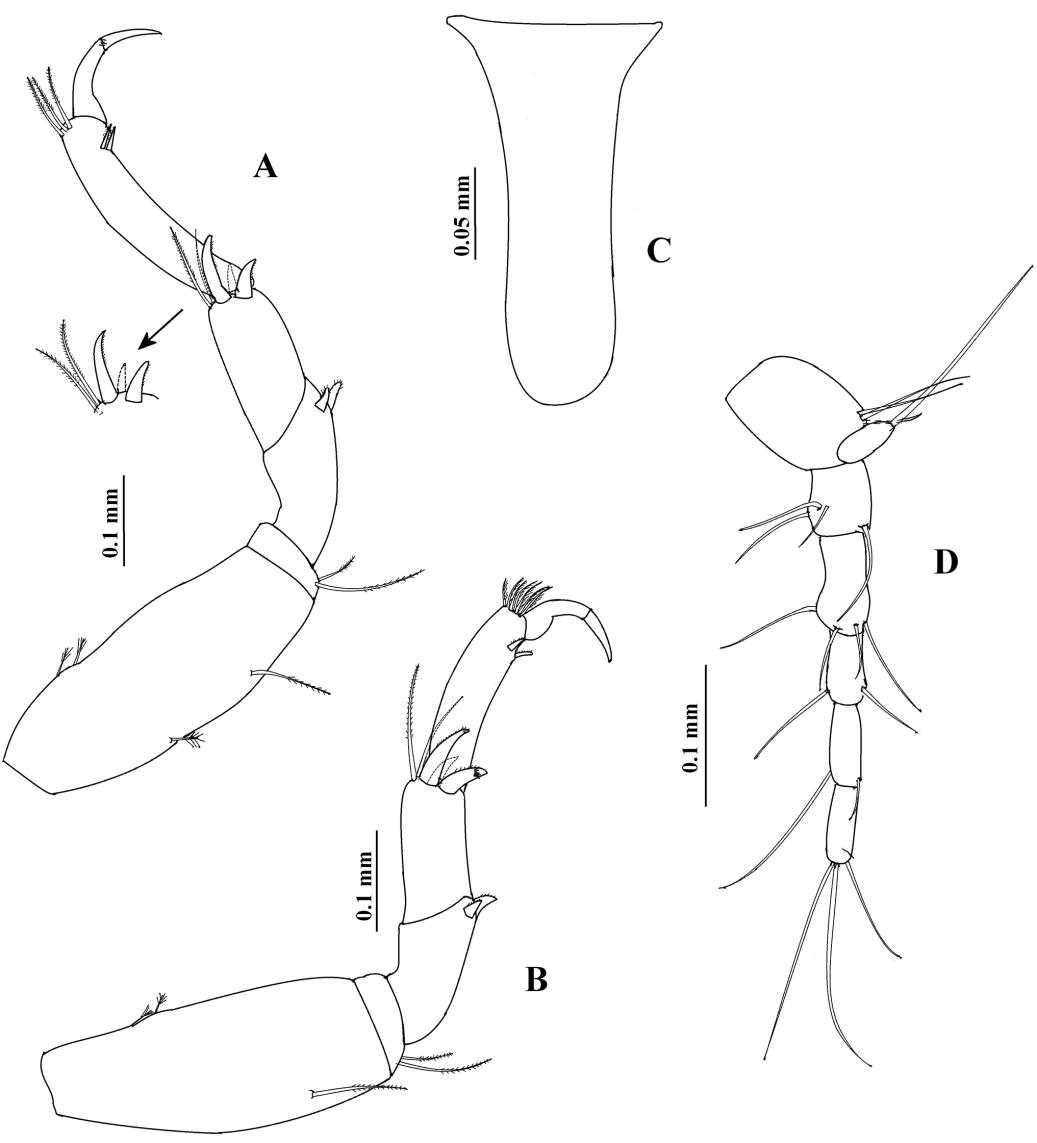
*Hargeria chetumalensis* sp. nov. Holotype ECOSUR0209, adult male, 2.9 mm. (A) Pereopod 5. (B) Pereopod 6. (C) Anal plate. (D) Uropod.

Pereopod 5 (Fig. 9A) similar to pereopod 4, but basis 2.6 times as long as broad, with one ventrolateral serrate seta and three plumose sensory setae on proximal half. Serrate setae on carpus not extending beyond median length of propodus. Propodus with three dorsodistal serrate setae.

Pereopod 6 (Fig. 9B) similar to pereopods 4 and 5, but basis 2.3 as long as broad. Merus as long as broad. Propodus 3.9 times as long as broad, with seven short dorsodistal serrate setae.

Uropod (Fig. 9D) with three dorsal setae on peduncle. Exopod uniarticulate, 0.8 times as long as first article of uropodal endopod, with three distal setae (two missing). Endopod with five articles; first, fourth, and fifth article of similar length; second article longer than other articles; with four distal setae; third article shorter than other articles; last article with three distal setae.

**Figure 10:**
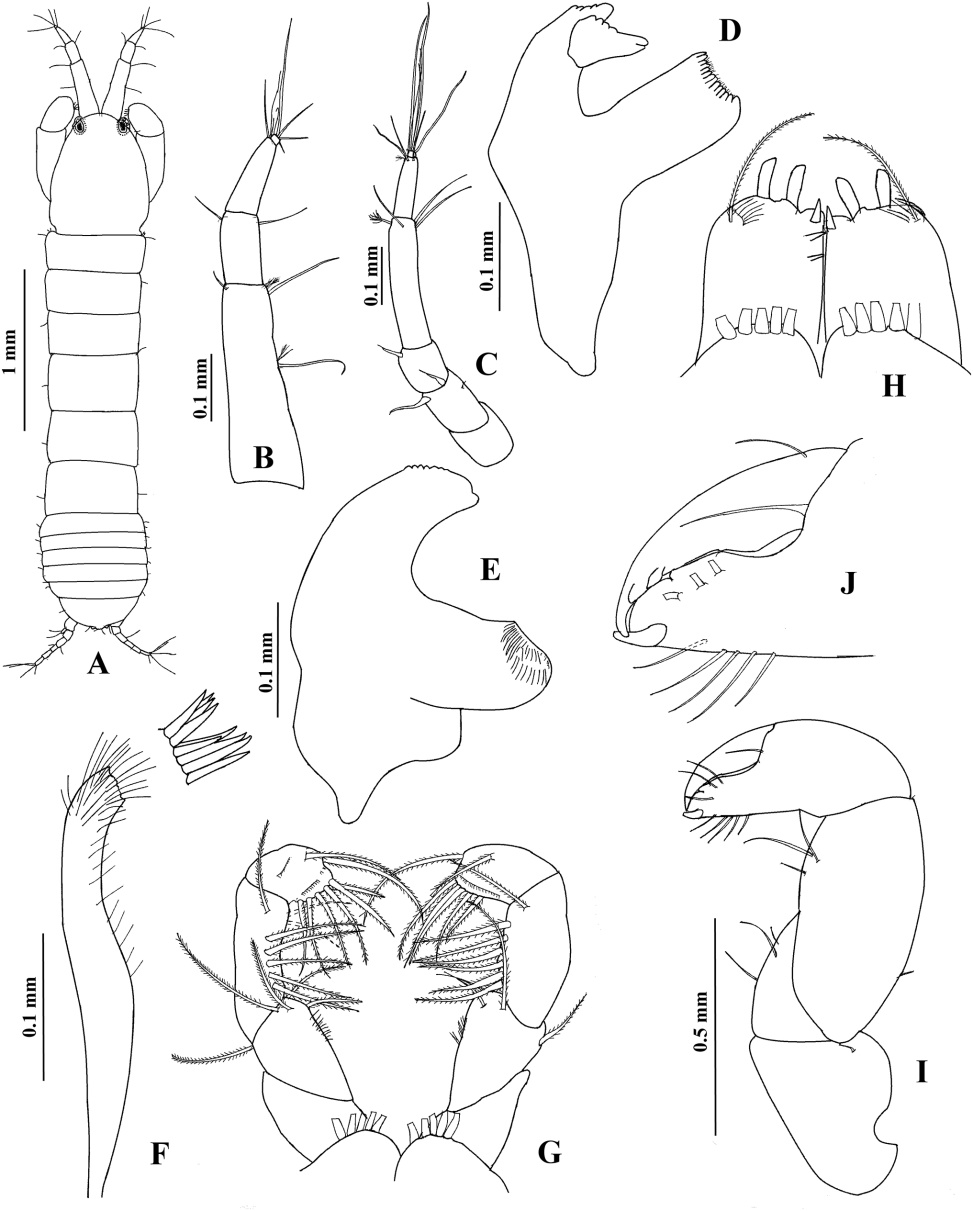
*Hargeria chetumalensis* sp. nov. Paratype ECOSUR0210, non-ovigerous female, 3.1 mm. (A) Habitus. (B) Antennule. (C) Antenna. (D) Left mandible. (E) Right mandible. (F) Maxillule. (G) Maxilliped. (H) Maxillipedal endite. (I) Cheliped. (J) Details of propodius and dactylus.

**Non-ovigerous female**. Paratype ECOSUR0210, 3.1 mm (Fig. 10A). Body 4.9 times as long as broad. Cephalothorax oval, 1.2 times as long as broad, as long as first three pereonites combined. Pereon about twice as long as cephalothorax; pereonites 4 and 6 subequal in length; pereonite 5 shorter than other pereonites. Pleon less than 0.2 times the total body length. Pleotelson about 0.3 times as long as pleon; posterior end with eight setae, four of them small (Fig. 12B).

Antennule (Fig. 10B) with four articles. First article 2.7 times as long as broad, with three setae and three plumose sensory setae on distal half. Second article 0.4 times as long as first article, 1.9 times as long as broad, with two distal setae. Third article 0.4 times as long as first article, 2.5 as long as broad. Fourth article minute, with three distal setae and one aesthetasc.

Antenna (Fig. 10C). First article as long as broad, naked. Second article 1.4 times as long as broad, with two distal spines and small distolateral seta. Third article 1.2 times as long as broad, with one distal spine. Fourth article 4.7 times as long as broad, with three simple setae and one plumose sensory seta. Fifth article 0.5 times as long as fourth article, 4.3 times as long as broad, with two distal setae and one plumose sensory seta. Sixth article minute, with six distal setae.

Mouthparts. Left mandible, ventral view, (Fig. 10D) with incisor shorter than lacinia mobilis, with four small processes; lacinia mobilis with four small processes and bifurcate apex; molar cylindrical, thick, with triturative ridges. Right mandible (Fig. 10E) with incisor crenulate, with slightly bifurcate apex; molar with triturative ridges. Labium (not illustrated) distally setose, with scales. Maxillule (Fig. 10F) setose, with nine spines. Right maxilliped (Fig. 10G) with five serrate setae on basis; first palp article 1.6 times as long as broad, naked; second palp article twice as long as broad, with one dorsodistal serrate seta, four ventrodistal setae and small ventrolateral setae; third palp article 3.2 times as long as broad, with eight ventrolateral serrate setae; fourth palp article 2.4 times as long as broad, with nine ventrolateral serrate setae and scales; endite (Fig. 10H) with three flat spines (two of them rounded terminally, the subdistal smaller, pointed), one dorsodistal seta, coupling hooks and scales.

Cheliped (Figs. 10I–10J) 0.3 times as long as total body length. Basis 1.3 times as long as broad, with one dorsol seta. Merus longer than broad, with three ventral setae. Carpus twice as long as broad, with three ventrodistal setae and two dorsal setae. Propodus with seta near dactylus articulation, comb-row with four setae (not illustrated). Fixed finger with three weak processes on cutting edge; proximal small; with eight setae, five of them situated on ventral side. Dactylus with proximodorsal seta and three lamellae on ventral edge.

**Figure 11:**
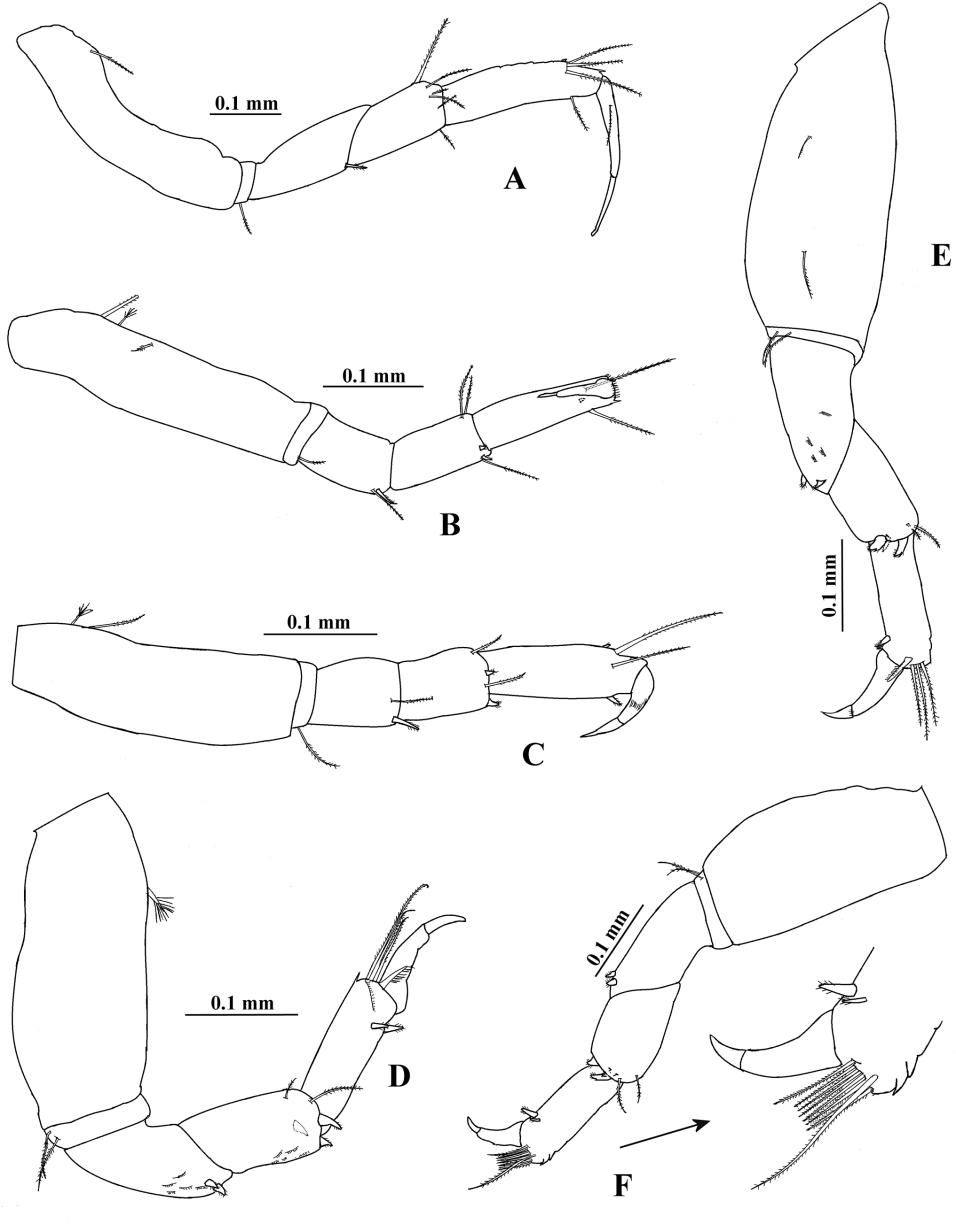
*Hargeria chetumalensis* sp. nov. Paratype ECOSUR0210, non-ovigerous female, 3.1 mm. (A) Pereopod 1. (B) Pereopod 2. (C) Pereopod 3. (D) Pereopod 4. (E) Pereopod 5. (F) Pereopod 6.

Pereopod 1 (Fig. 11A) with basis 4.3 times as long as broad, with one proximal serrate seta. Ischium with one serrate seta. Merus 1.3 times as long as carpus, 2.2 times as long as broad, with one small ventrodistal serrate seta. Carpus 2.4 times as long as broad, with six distal serrate setae. Propodus 3.8 times as long as broad, with distal serrate setae (three dorsal and one ventral). Dactylus and unguis combined as long as propodus; dactylus with one proximal serrate seta.

Pereopod 2 (Fig. 11B) with basis four times as long as broad, with one proximal serrate seta and two dorsodistal plumose sensory setae. Ischium with one serrate setae. Merus 0.9 times as long as carpus, 1.7 times as long as broad, with one ventrodistal serrate seta and one slender serrate spine. Carpus twice as long as broad, with three distal setae and two ventrodistal minute spines. Propodus 3.3 times as long as broad, with two dorsodistal serrate setae and small distoventral spine. Dactylus and unguis combined about 0.5 times as long as propodus.

Pereopod 3 (Fig. 11C) similar to pereopod 2, but basis three times as long as broad, with one dorsoproximal plumose sensory seta. Merus 1.2 times as long as broad. Carpus 1.4 times as long as broad.

Pereopod 4 (Fig. 11D) with basis 2.4 times as long as broad, with one dorsal plumose sensory seta. Ischium with two serrate setae. Merus 1.2 times as long as carpus, 2.3 times as long as broad, with two ventrodistal serrate spines and scales. Carpus twice as long as broad, with two distal serrate setae, three serrate spines and scales. Propodus 3.3 times as long as broad, with three dorsodistal serrate setae, one pinnate seta, two ventrodistal serrate spines and scales. Dactylus and unguis combined 0.7 times as long as propodus.

Pereopod 5 (Fig. 11E) similar to pereopod 4, but basis with two dorsal serrate setae. Merus 1.9 times as long as broad.

Pereopod 6 (Fig. 11F) similar to pereopods 4 and 5, but basis apparently naked. Carpus 1.7 times as long as broad. Propodus 2.8 times as long as broad, with eight dorsodistal serrate setae (one longer) and two ventrodistal serrate spines.

**Figure 12:**
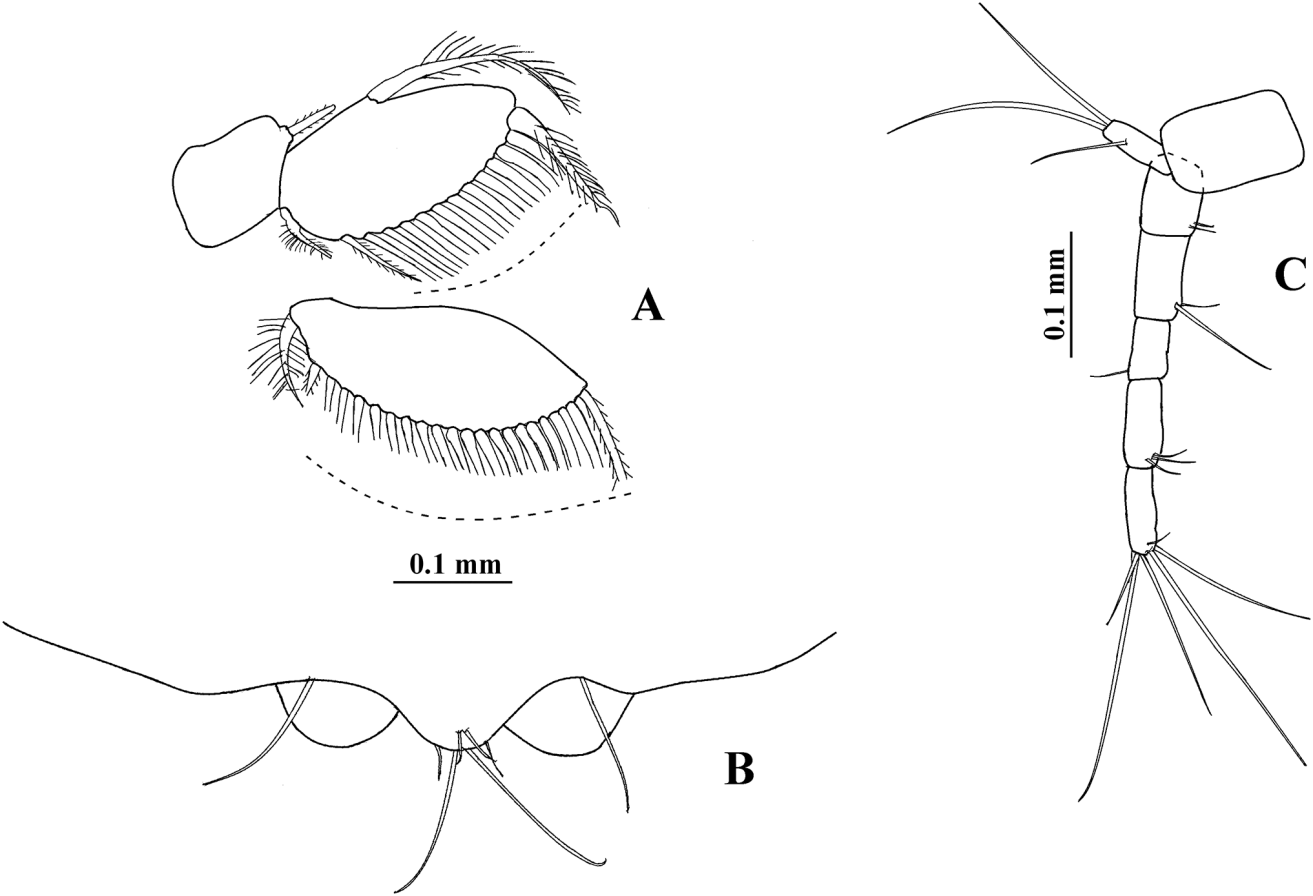
*Hargeria chetumalensis* sp. nov. Paratype ECOSUR0210, non-ovigerous female, 3.1 mm. (A) Pleopod. (B) Posterior end of pleotelson. (C) Uropod.

Pleopod 1 (Fig. 12A) with one serrate seta on peduncle. Exopod with one proximoventral circumplumose seta and 22 ventral setae. Endopod with one dorsal circumplumose outer seta, one short proximoventral circumplumose inner seta, and 13 ventral setae.

Uropod (Fig. 12C) with naked peduncle. Exopod uniarticulate, 0.9 as long as first endopodal article, with three setae. Endopod with five articles; first article shorter than second article, with two distal setae; third article shorter than other articles, with one distal seta; fourth and fifth articles of subequal length, with three and six distal setae, respectively.

**Type locality.** Raudales, Guerrero Lagoon, Quintana Roo, Mexico.
**Distribution.** Chetumal Bay and Guerrero Lagoon Bay System, Quintana Roo, Mexico.
**Habitat.** Shallow-water in rocks with sediment and alga *Batophora* sp.

**Remarks.** Comparing males of *H. chetumalensis* sp. nov. with *H. rapax*, interspecific differences can be seen in the rostrum, chelipeds, antennules, antennae, mandibles, pereopods (1, 2, 4, 6) and the anal plate. For example, males of *H. chetumalensis* have a pointed, narrow rostrum that does not extend beyond the eyes; in contrast, *H. rapax* has a convex and broad rostrum that does extend beyond eyes. *H. chetumalensis* has the first peduncle article of the antennule more than eight times as long as broad, while in *H. rapax* it is up to seven times as long as broad; *H. chetumalensis* has the carpus of cheliped about eight times as long as broad, whereas in *H. rapax* it is five to seven times as long as broad. Also, in *H. chetumalensis* the two long distal setae on merus of pereopod 4 exceed the median length of propodus, while in *H. rapax* such setae do not. Furthermore, *H. chetumalensis* has a tongue-shaped anal plate, which is more than three times as long as broad, whereas *H. rapax* has a capitate anal plate that is less than three times as long as broad (other differences see Table 2).

**Table 2:** Main differences between males of *H. rapax* and *H. chetumalensis* sp. nov.

	*H. rapax*	*H. chetumalensis* sp. nov.
Total length (mm)	2.3–2.6	1.9–2.9
Rostrum length vs eyes	Exceeding	Not exceeding
Fourth article, antenna	Up to 4.7 times as long as broad	Up to 6.6 times as long as broad
Chelipedal carpus	Five to seven times as long as broad	About eight times as long as broad
Proximal process, fixed finger of cheliped	Pointed	Rounded
No. distal setae on merus, pereopod 1	Two simple	One serrate
No. distal setae on carpus, pereopod 1	Six simple	Three serrate
Propodus, pereopod 1	Up to 3.9 times as long as broad	Up to 5.6 times as long as broad
No. setae on merus, pereopods 2 and 3	One simple	0
No. spines on merus, pereopods 2 and 3	One simple	One serrate
No. setae on propodus, pereopod 6	Eight simple	Seven serrate
Shape of anal plate	Capitate	Tongue-shaped

Regarding females, *H. chetumalensis* is stouter, the body is 4.9 times as long as broad, unlike *H. rapax* where the body is 6.2 times as long as broad; *H. chetumalensis* has the first three pereonites of similar length, whereas in *H. rapax* only the second and third pereonite are similar. Also, *H. chetumalensis* has three lamellae on the cutting edge of the cheliped dactylus, while *H. rapax* has eight. Furthermore, *H. chetumalensis* has the third maxillipedal article 3.2 times as long as broad, whereas in *H. rapax* it is 2.1 times as long as broad (other differences see Table 3).

**Table 3:** Main differences between females of *H. rapax* and *H. chetumalensis* sp. nov.

	*H. rapax*	*H. chetumalensis* sp. nov.
Total length (mm)	2.9–3.7	3.1
First antennular article	Up to 3.7 times as long as broad	Up to 2.7 times as long as broad
Fourth antennal article	Up to 3.3 times as long as broad	Up to 4.7 times as long as broad
Fifth antennal article	Up to 2.2 times as long as broad	Up to 4.3 times as long as broad
No. small processes on incisor, left mandible	3	4
No. inner lamellae on chelipedal dactylus	8	3
No. distal setae on merus, pereopod 1	Two to three simple	One serrate
No. setae on merus, peropod 2 and 3	Two simple	One serrate
No. spines on merus, peropods 2 and 3	0	One serrate

### Molecular analyses

The morphological differences found between *H. rapax* and *H. chetumalensis*, and also between *Hargeria*, *Chondrochelia*, and *Leptochelia* genera are supported by the molecular analyses based on the COI (Fig. 15). Our results show a genetic divergence of 14.7% between *H. rapax* and *H. chetumalensis*, which according to Costa et al. (2007) and Montiel-Martínez et al. (2008), exceeds the lowest value (4.9%) of interspecific divergence found in crustaceans. Also, the intraspecific divergence among the populations of *H. rapax* along the coast of the USA was 1.2% (*n* = 5), while in Mexico (for *H. chetumalensis*) it was 0.2% (*n* = 21). It is probable that the larger genetic divergence between the populations of Florida and Maryland is due to greater geographic distance between them than those found in the Chetumal Bay and Guerrero Lagoon System.

On the other hand, with regards of genetic divergence between *Hargeria* species and species of the other genera analyzed, both species of *Hargeria* have a genetic divergence higher to 40% concerning *C. dubia* and 35–38% with *Leptochelia* species. These values are higher than those considered for intrageneric divergence on Crustacea (19–32%) (Costa et al., 2007; Matzen Da Silva et al., 2011). Our molecular and morphological data support Guţu’s proposal to recognize *Hargeria* as a valid genus.

### Morphological analyses of setal and cuticular structures

**Figure 13:**
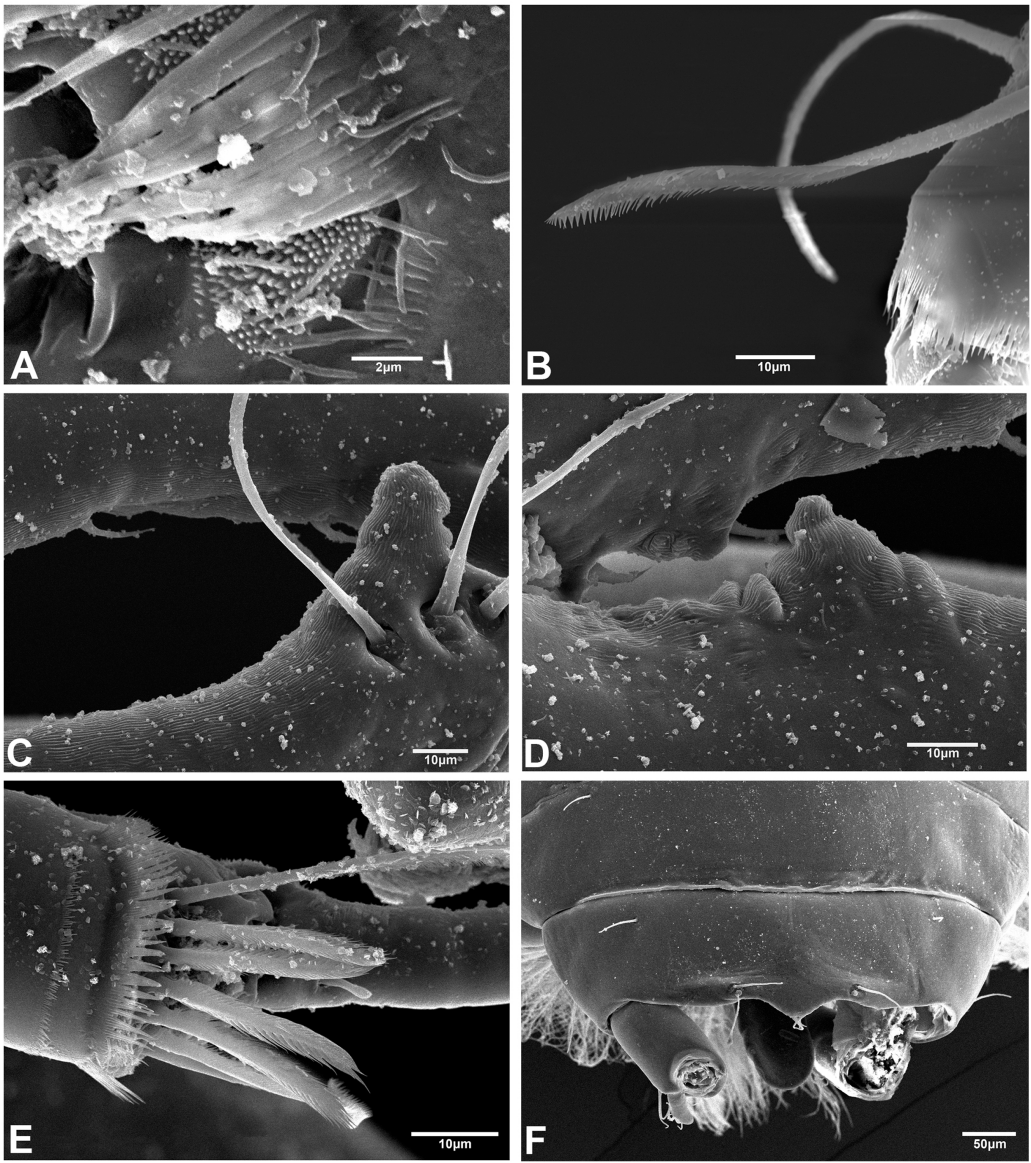
*Hargeria chetumalensis* sp. nov. males from Raudales, Quintana, Roo. SEM images. Males, 2.4 mm, ECOSUR. (A) Pores and scales on propodus, pereopod 1. (B) Serrate setae with terminal pore, pereopod 2. Male, 1.9 mm, ECOSUR. (C) Distal chelipedal process. (D) Proximal chelipedal processes. (E) Serrate setae on propodus, pereopod 6. Male, three mm. ECOSUR. (F) Pleotelson, uropods broken.

**Figure 14:**
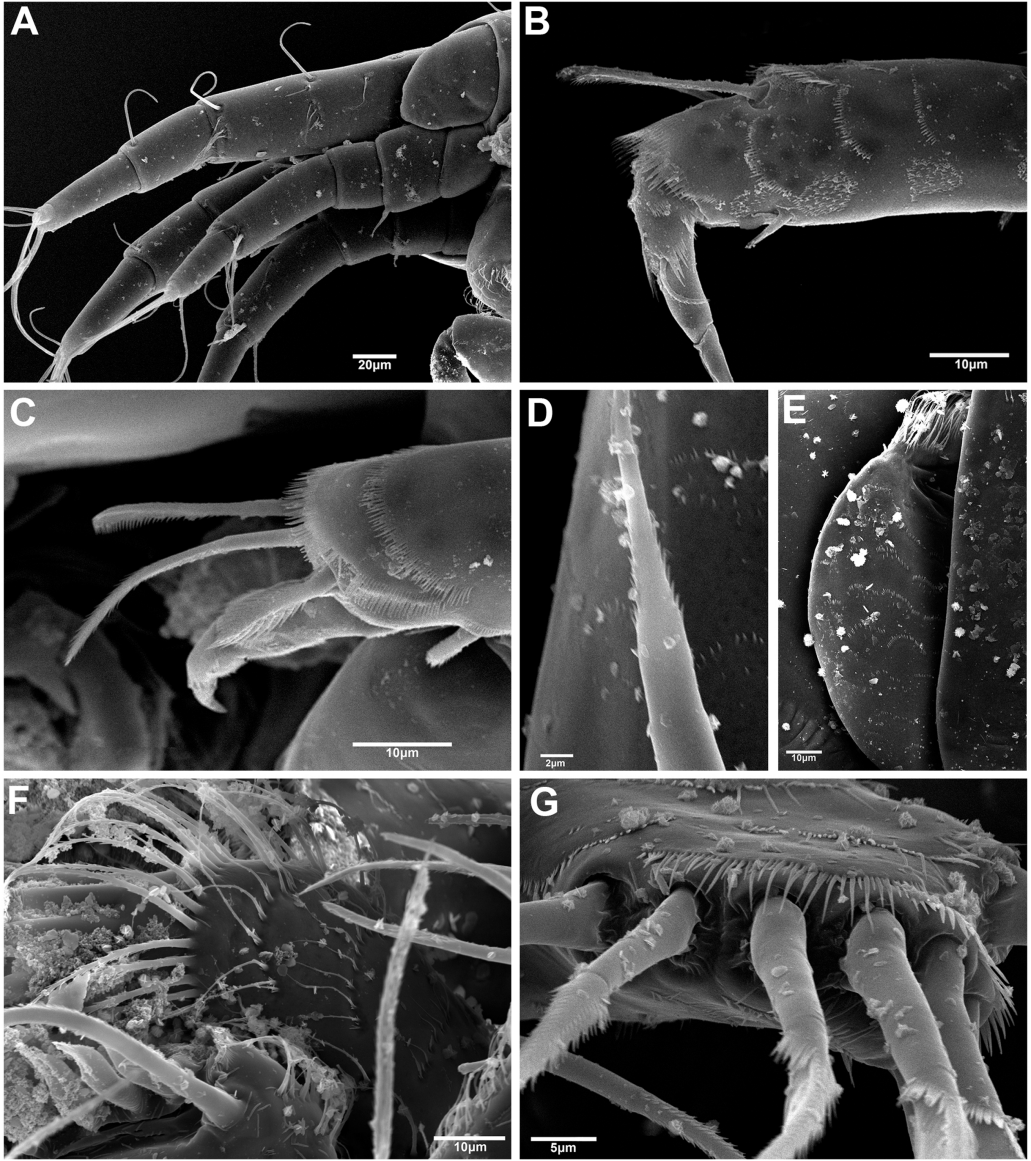
*Hargeria chetumalensis* sp. nov. manca and female from Raudales, Quintana, Roo. SEM images. Manca, 0.9 mm, ECOSUR. (A) Antennules and antennae, lateral view. (B) Serrate setae, scales and pores on propodus, pereopod 2. (C) Serrate and plumose sensory setae, spinulate scales and pores on propodus, pereopod 5. Non-ovigerous female, 2.7 mm, ECOSUR. (D) Serrate spiniform seta on second antennal article. (E) Scales and serrate setae on labium. (F) Serrate setae and plumose sensory setae in maxillule. (G) Scales and serrate setae on maxillipedal fourth article.

The SEM analysis showed that the cuticle of all structures, as well as setae of pereopods and mouthparts are more ornate than they can appear using the optical microscope, where they may appear to be simple; however, they comprise scales, pores, and small setae, giving the “serrated” appearance on such features. For example, Fig. 13 shows the details of pores, scales, and serrated setae on the propodus of pereopods 1, 2, and 6 (male, 2.4 mm); the processes and cutting edge of the cheliped fixed finger (male, 1.9 mm); as well as the pleotelson and anal plate (male, three mm) of *H. chetumalensis*. With regards to female and manca, Fig. 14 shows the seta, spiniform setae and scales of antennules and antennae (manca, 0.9 mm, Fig. 14A; non-ovigerous female, 2.7 mm, Fig. 14D); pereopods (manca, Figs. 14B and 14C), and mouthparts (non-ovigerous female, Figs. 14E–14G). It is important to note that these features are present in all life stages, from manca to adult.

It is the first time that this type of ornamentation of the cuticle and setae is reported for species belonging to the Leptocheliidae. Therefore, we consider that a more detailed examination of these structures will allow us a better characterization of all structures. With this kind of information, we will have the robustness to clarify the morphological differences of the supposed species complexes; furthermore, we will obtain more information for phylogenetic studies.

## Discussion

**Figure 15:**
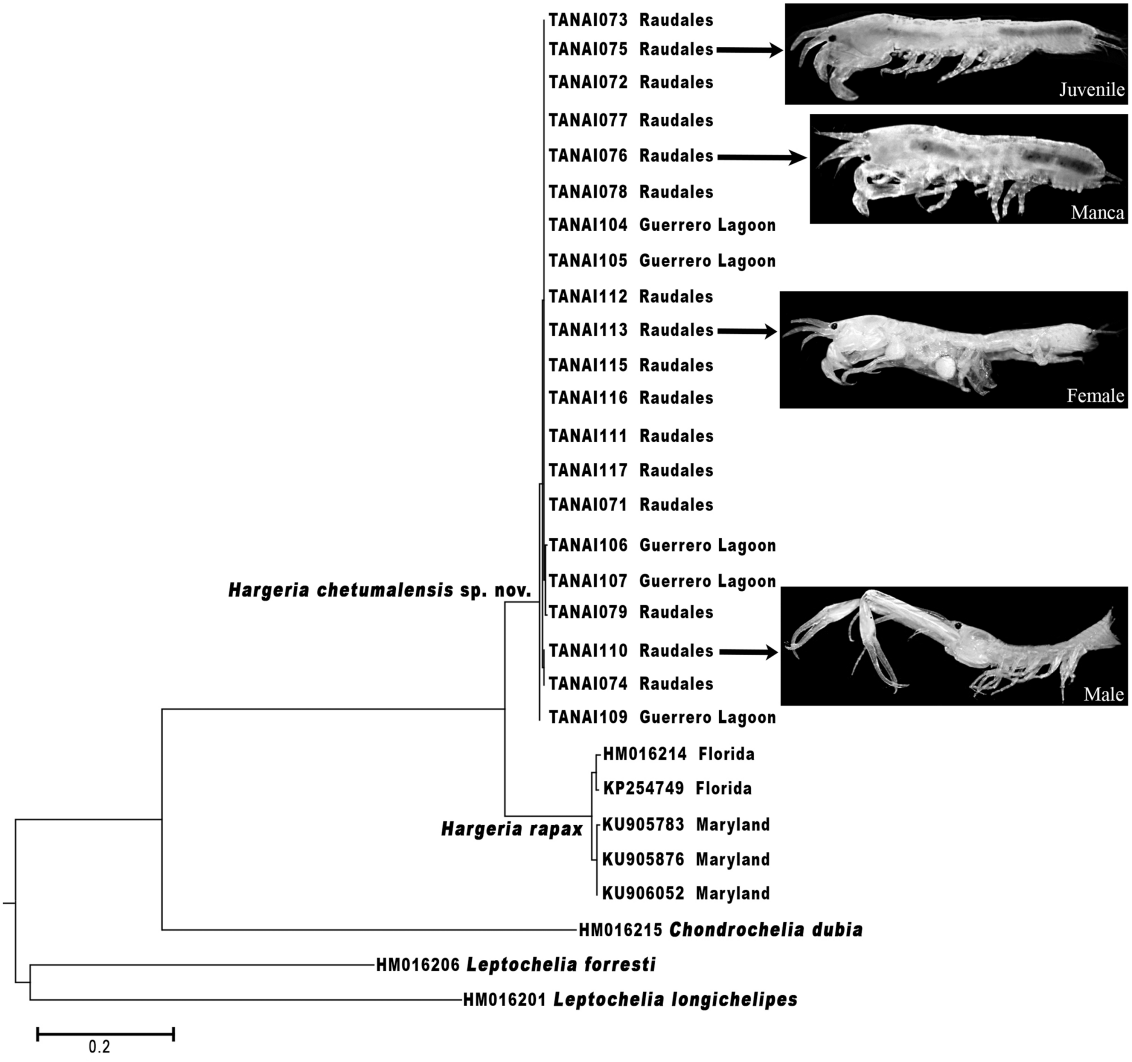
Maximum likelihood tree of COI sequences. Using Hasegawa–Kishino–Yano with a discrete Gamma distribution with five rate categories and by assuming that a certain fraction of sites is evolutionarily invariable (HKY+G+I).

The use of the integrative taxonomy in tanaids, according to Larsen (2001), Bamber (2010), Drumm (2010), and Larsen & Froufe (2010, 2013), is decisive in demonstrating the differences among cryptic species; i.e., discriminating new species or reinstating those species considered as junior synonyms. Furthermore, according to Błażewicz-Paszkowycz et al. (2014) the use of molecular technics is advantageous to link the different stages of life in tanaidaceans. When we analyzed the genetic sequences of *H. chetumalensis* sp. nov. and *H. rapax*, the data allowed us to confirm that the morphological features used for the recognition of both species were adequate to link males, females, and juvenile stages, although these species have a strong polymorphism (Fig. 15).

Besides, the use of the morphological and molecular information is advantageous to determine, with some reliability, the geographic range of the species. On the evidence provided in this study, *H. rapax* is restricted to the east coast of the USA, while *H. chetumalensis* is only known in the eastern part of the Chetumal Bay and Guerrero Lagoon, Mexico.

As we noted above, *H. rapax* constitute a cryptic species along the Northwestern American Atlantic. Due to the ecological conditions (e.g., ph, oxygen, and salinity concentrations, current velocity) along the extensive coastline varies considerably, it would be unlikely that an estuarine species could have a wide distribution. A detailed revision of the other specimens reported in this region as *H. rapax* need to be revaluated to corroborate this idea; however, it is beyond the scope of this study.

## Conclusions

The taxonomy of tanaidaceans represents a great challenge, since their small size (one to three mm on average), high intraspecific polymorphisms, and the lack of specialized regional literature have increased the degree of complexity for the recognition of genera and species. In recent years, the use of morphology, genetic, and morphometric data have allowed to detect and solve some taxonomic problems related to cryptic species of leptocheliids. Herein, based on morphological and molecular data, we give new evidence to support the recognition of *Hargeria* as a valid genus, different from *Chondrochelia* and *Leptochelia*. Besides, we redescribed *H. rapax* based upon type and topotype materials; also, we described the second species belonging to *Hargeria*, which is the first tanaidacean described from the Mexican Caribbean. We consider that the information provided in this work will help as the baseline to clarify the taxonomic status of the specimens that have been reported under the name of *H. rapax* within the wide geographical distribution that was attributed to the species.

## Supplemental Information

10.7717/peerj.7472/supp-1 Supplemental Information 1 COI sequences of *Hargeria chetumalensis* sp. nov.

## Additional Information and Declarations

### New Species Registration

The following information was supplied regarding the registration of a newly described species: Publication LSID: urn:lsid:zoobank.org:pub:B55A046E-55AA-491A-ABED-C9BA3D7300DE *Hargeria chetumalensis* LSID: urn:lsid:zoobank.org:act:A8D8DCA7-E71C-4124-83B7-F5E66CDA31A1.

### DNA Deposition

The following information was supplied regarding the deposition of DNA sequences: The COI sequences of *Hargeria chetumalensis* sp. nov. are available within the project Tanaidacea from Mexico (TANME) at Barcode of Life Data System (http://www.boldsystems.org/): TANAI071-15, TANAI072-15, TANAI073-15, TANAI074-15, TANAI075-15, TANAI076-15, TANAI077-15, TANAI078-15, TANAI079-15, TANAI104-15, TANAI105-15, TANAI106-15, TANAI107-15, TANAI109-15, TANAI110-15, TANAI111-15, TANAI112-15, TANAI113-15, TANAI115-15, TANAI116-15, TANAI117-15. The sequences of *Hargeria rapax* (HM016214, KP254749, KU905876, KU905783, KU906052), *Chondrochelia dubia* (HM016215), *Leptochelia forresti* (HM016206), and *Leptochelia longichelipes* (HM016201) are available at GenBank.

### Data Availability

The following information was supplied regarding data availability: The specimens reviewed of *Hargeria rapax* are deposited in the collection of the National Museum of the Natural History, Smithsonian Institution, Washington, USA (USNM 32176, USNM 101781, USNM 65779). The specimens of *Hargeria chetumalensis* are deposited in the Reference Collection of Benthos, El Colegio de la Frontera Sur, Chetumal, Mexico (ECOSUR 0209, ECOSUR 0210, ECOSUR 0211, ECOSUR 0212, ECOSUR-C1079, ECOSUR-C1080). The raw data of COI sequences from *Hargeria chetumalensis* sp. nov. (sequence data, electropherograms, trace files, primer details, photographs, life stages, and collection localities) are available within the Project “Tanaidacea from Mexico” (TANME) at Barcode of Life Data System (http://www.boldsystems.org/): TANAI071–079, TANAI104–107, TANAI109–113, TANAI115–117.
